# Optimising measurement of health-related characteristics of the built environment: Comparing data collected by foot-based street audits, virtual street audits and routine secondary data sources

**DOI:** 10.1016/j.healthplace.2016.10.001

**Published:** 2017-01

**Authors:** Triantafyllos Pliakas, Sophie Hawkesworth, Richard J. Silverwood, Kiran Nanchahal, Chris Grundy, Ben Armstrong, Juan Pablo Casas, Richard W. Morris, Paul Wilkinson, Karen Lock

**Affiliations:** a Faculty of Public Health and Policy, London School of Hygiene and Tropical Medicine, London, UK; b Faculty of Epidemiology and Population Health, London School of Hygiene and Tropical Medicine, London, UK; c Faculty of Population Health Sciences, University College London, UK; d School of Social and Community Medicine, University of Bristol, Bristol, UK

**Keywords:** Built environment, Research design, Environment measurement, Health

## Abstract

The role of the neighbourhood environment in influencing health behaviours continues to be an important topic in public health research and policy. Foot-based street audits, virtual street audits and secondary data sources are widespread data collection methods used to objectively measure the built environment in environment-health association studies. We compared these three methods using data collected in a nationally representative epidemiological study in 17 British towns to inform future development of research tools. There was good agreement between foot-based and virtual audit tools. Foot based audits were superior for fine detail features. Secondary data sources measured very different aspects of the local environment that could be used to derive a range of environmental measures if validated properly. Future built environment research should design studies *a priori* using multiple approaches and varied data sources in order to best capture features that operate on different health behaviours at varying spatial scales.

## Highlights

• This study compares multiple data collection methods for measuring built environment features.
• Virtual street audits are reliable for more objective built environment measures.
• Street-based audits are superior for collecting fine detail environmental features.
• Routine secondary data sources need less resources but must be properly validated.
• Appropriate methods for health studies vary depending on the research question and resources.

## 1 Introduction

The role of the neighbourhood built environment in influencing diet, physical activity and health outcomes across the life course has received considerable attention in public health research and policy (Bader et al., 2010, Calogiuri and Chroni, 2014, Caspi et al., 2012, Charreire et al., 2010, Charreire et al., 2014, Christian et al., 2015, de Vet et al., 2011, Ding and Gebel, 2012, Ding et al., 2011, Dunton et al., 2009, Goodwin et al., 2013, King, 2015, Saelens and Handy, 2008, World Health Organization, 2010). The built environment has been defined as the physical environment constructed by human activity (Saelens and Handy, 2008), and built environment-health research ideally aims to capture and understand the impact of both contextual (i.e. nature of the area such as access to services) and compositional factors (i.e. nature of residents reflecting the collective social functioning of an area) on people's health behaviours (Cummins et al., 2007, Macintyre, 2007, Macintyre et al., 2002).

Potentially relevant built environment factors have been studied using objective measures involving the use of primary and secondary spatial data (Thornton et al., 2011) and there is an extensive body of research using a range of different data collecting approaches (Brownson et al., 2009, Charreire et al., 2010, Krenn et al., 2011, Schaefer-McDaniel et al., 2010). Much research in this area was initially driven by the availability of secondary data (Macintyre et al., 2002). Such routine data combined with Geographical Information Systems (GIS) can be used to construct environmental measures, including density and spatial availability, walkability indices and undertake spatial analysis and modelling to examine the impact of the neighbourhood environment on people's health behaviours and outcomes (Burgoine et al., 2013, Caspi et al., 2012, Leslie et al., 2007). However, routinely available spatial data have well-recognised limitations, including problems with the use of administrative boundaries to define neighbourhoods and the limited types of environmental exposures that can be investigated (Cummins et al., 2005, Lucan, 2015). Secondary data sources may also give rise to issues of specificity (i.e. the proportion of shops that are correctly identified as being specific types for an analysis e.g. retail food outlets (Fleischhacker et al., 2013)) and misclassification of environmental exposures. When possible, neighbourhood audits should be conducted to confirm the validity of routine data sources (Cummins and Macintyre, 2009, Fleischhacker et al., 2013, Lucan et al., 2013, Pliakas et al., 2014).

Systematic neighbourhood audits using foot-based or virtual street audit tools, such as Google Street View (GSV) and Bing Maps (or Microsoft Virtual Earth), have been used to collect primary data on factors theoretically relevant but not available in existing routine data (Brownson et al., 2009, Shareck et al., 2012, Wu et al., 2014). The majority of tools available, especially using GSV, have been developed for specific North American towns (Charreire et al., 2014) or for the UK (Griew et al., 2013, Wu et al., 2014). Neighbourhood audits involve direct on-foot or virtual observations by trained observers who use checklists to observe and rate physical and social attributes of neighbourhoods. The geographic unit of recorded observation in audits is the face block (e.g. the block segment on one side of a street (Clarke et al., 2010; Sampson and Raudenbush, 1999)) or street segment (Charreire et al., 2014). Street segment measures are typically developed in GIS by dividing the street network within the study area into road sections termed ‘links’ (Bethlehem et al., 2014, Griew et al., 2013) or by generating intersection to intersection segments (Badland et al., 2010). Some current audit instruments can be found at http://activelivingresearch.org/. Designing systematic audit tools that have measurement validity, reliability and specificity relevant to both health outcomes of interest and the context of a study have been identified as an important area of methodological research (Shareck et al., 2012, Zenk et al., 2007).

Using GSV to conduct virtual street audits is easy, cheap and safe as well as being transparent as it is available to the general public, public health and planning researchers and practitioners (Charreire et al., 2014). Systematic, foot-based, street audits are relatively expensive and time consuming (Badland et al., 2010, Ben-Joseph et al., 2013, Wu et al., 2014). The growing use of GSV audits to capture exposures relevant to physical activity and food environments have prompted some studies to conduct comparisons with foot-based audits (Charreire et al., 2014, King, 2015). However, the majority of these comparisons have focused on a limited number of environmental dimensions (Charreire et al., 2014).

Detailed data collected by direct observation can produce valuable information for those who can act on the findings, such as urban and transport planners and policy makers (Brownson et al., 2009). Most foot-based audit studies have focused on single risk factors, e.g. physical activity (Lee et al., 2005, Pikora et al., 2002) or diet (Glanz et al., 2007, Saelens et al., 2007) or occasionally a combination of the two (Bethlehem et al., 2014), despite the complex, multifactorial aetiology of cardiovascular and other chronic diseases. To date there are no tools simultaneously capturing dimensions of the built environment that may be relevant for influencing multiple health behaviours (e.g. diet, physical activity and alcohol intake) that together contribute to improving complex population health outcomes such as obesity.

This paper aims to explore objective measurement approaches for health related aspects of the built environment by comparing built environment data captured by secondary data sources, foot-based and GSV audits. To our knowledge, no other study has simultaneously compared primary data from foot-based audits with remote-sensing virtual street audits and secondary data sources. This methodological comparison aims to enable researchers to make better informed decisions in the design and analysis of large scale epidemiological studies of the effect of the built environment on health outcomes.

## 2 Methods

### 2.1 Audit tools

We developed a new instrument, the ‘Older People's Environments and CVD Risk’ (OPECR) tool, initially as a foot-based audit tool to capture detailed features of the local environment particularly relevant to older people's health behaviours. OPECR was designed as a data collection pro-forma document (i.e. paper form) to collect geographical data relevant to older people's behaviours by direct observation of local neighbourhoods. The tool consists of 100 indicators, including density measures (i.e. density of food shops and alcohol outlets), price and availability of selected food, alcohol and tobacco products, measures of “walkability” of the environment for older people (e.g. connectivity of streets, road speed, traffic volume, quality of pavements and pedestrian crossings), transport accessibility and connectivity (e.g. bus stops and routes) and land use mix. The audit tool is available as supplementary material (Appendix S1). The comparisons presented here are nested within a wider study of the association between aspects of the neighbourhood environment and physical activity, dietary behaviours and cardiovascular disease risk in older adults in 20 UK towns (Hawkesworth et al., 2015).

The OPECR tool was modified to assess neighbourhood environments remotely using GSV to allow for the comparison of the two techniques. Only minimal adaptations of the street-audit tool were required as it was still possible to assess the majority of environmental features virtually. Information on prices in shops, traffic volume and litter were removed, whilst variables to capture the quality and date of the GSV image were added.

### 2.2 Primary data collection

Fieldworkers were recruited to conduct foot-based audits in 20 towns across the UK (17 in England and 3 in Scotland) that were included in two national cohort studies, the British Regional Heart Study (BRHS) (Walker et al., 2004) and the British Women's’ Heart and Health Study (BWHHS) (Lawlor et al., 2003). Fieldworkers were fully trained in the use of the OPECR tool and supervisors conducted frequent field visits in each town to ensure data collection quality. A street segment was the unit of data collection and was defined as the length of a road that does not change in name or distinctly in character. The start and end point of the segment were recorded and these were used as reference points for the GSV audit. The lower layer super output area (LSOA) was used to draw maps of the audited areas using Google Maps.

We piloted the audit tool in two towns, Bristol and Guildford, in September-October 2009. Fieldworkers were asked to conduct concurrent independent repeat audits of specific segments in order to assess inter-rater reliability. Data collection for the foot-based audits for the remaining towns took place between October 2011 and September 2014. Fieldworkers worked in pairs and recorded all relevant aspects of the OPECR tool for both sides of the segment. All street segments were audited in all LSOAs in study towns where study subjects lived. Foot-based audit data were entered into an Access database before being exported into Stata 14.1 (StataCorp LP, 2015).

The GSV audit was conducted in a subset of two study towns (Ipswich and Newcastle-under-Lyme) chosen from the original 20 because they were towns of similar population size located in different English geographical regions with different deprivation rankings (Index of Multiple Deprivation 2010) to increase the variability of the environmental data. In 2012, Ipswich, located in east England, had a population of 134,500 and was ranked 87 out of 326 English local authorities in terms of area deprivation, while Newcastle-under-Lyme, located in north west England, had a population of 124,000 and was ranked 152 in terms of area deprivation (Department for Communities and Local Government, 2013). Foot-based audits in Ipswich were conducted between October 2011 and December 2011 and in Newcastle-under-Lyme between November 2013 and December 2013. GSV audits in Newcastle-under-Lyme were conducted between May 2014 and August 2014 and in Ipswich between December 2013 and May 2014. In Ipswich, the majority of GSV imagery was uploaded seven months after the foot-based audits. The time difference between foot-based audits and GSV imagery upload in Newcastle-under-Lyme was between 1 and 4 years.

Two fieldworkers, independent of those collecting foot-based audit data, were trained in the data collection procedure using GSV. The GSV fieldworkers were unable to access foot-based audit data so that GSV data collection was completely independent. Street segments in GSV were defined by matching the start and end points of segments audited in the foot-based audits. The fieldworkers virtually ‘walked through’ the street segments, first recording one side and then the other side of the segment. GSV audit data were entered into an Access database before being exported into Stata 14.1 (StataCorp LP, 2015).

### 2.3 Secondary data collection

Selected relevant measures of the physical environment at the LSOA level in the 17 English towns were generated from secondary data sources using ArcGIS 10.3 (ESRI, 2014). Seven environmental variables were examined that were treated as *a priori* directly comparable and proxy variables for features collected by foot-based audits. These related to freely available secondary data sources on transport, access to services, green space and land use mix. Transport related variables included annual average daily flow (AADF) for all motor vehicles and counts of bus stops in 2014. An AADF is the average over a full year of the number of vehicles passing a point in the road network each day. An AADF is measured in major roads (Motorway and A-class roads) and minor roads (B-roads, C-roads and unclassified roads). The raw manual counts are collected by trained enumerators over a period of one hour (Department for Transport, 2015). Data on the road network (motorways, A-roads and total road network) were obtained from Digimap Meridian 2 National (Digimap Ordnance Survey, 2015). Data on road traffic injuries for 2014 were obtained from the Department for Transport. Location of bus stops and stations was available through the National Public Transport Access Nodes database (Department for Transport, 2014). Location of General Practices and dental surgeries (in 2006), and pharmacies (in 2004) was available through the Neighbourhood Statistics website (Office for National Statistics, 2015). Data on the percentage of area that is public green space in LSOA was available through the Generalised Land Use Database Statistics for England (in 2005) (Office for National Statistics, 2015). Using the same database we constructed a spatial entropy score (SENS) using four different land types; residential, non-residential, green space and other. The SENS is calculated using the formula

$$\mathrm{SENS}=\sum \frac{(P_{i}*\ln P_{i})}{\ln N}$$

where *P* is the proportion of each land type *i* in the LSOA and *N* is the number of land types. The SENS is a measure of evenness and ranges from 0 to 1 with higher values representing a more heterogeneous and evenly mixed land-use per LSOA (Pliakas et al., 2014).

### 2.4 Statistical methods

Inter-rater reliability (IRR) was assessed for both the foot-based and GSV audit tools. Agreement was assessed for two independent observers for each segment covered by the analysis. The kappa statistic was used to assess inter-rater agreement for categorical variables (Armstrong et al., 1992, Fleiss, 1981, Landis and Koch, 1977) and intraclass correlation coefficient (ICC) for one-way random effects model (Shrout and Fleiss, 1979) was calculated to assess agreement for continuous variables including counts. Bias-corrected confidence interval for the kappa statistic was calculated using 1000 bootstraps. Categories for agreement level suggested by Landis and Koch (Landis and Koch, 1977) were used to describe kappa statistics 0.80–1.00, almost perfect; 0.60–0.79, substantial; 0.40–0.59, moderate; 0.20–0.39, fair; and 0.00–0.19, poor) and values suggested by Cicchetti (Cicchetti, 1994) were used for ICC 0.75–1.00, excellent; 0.60–0.74, good; 0.40–0.59, fair; and 0.00–0.39, poor). Agreement and IRR was assessed for all individual and composite (eg. shops and services or any amenities) audit variables in their original scale and in the format in which they were collected.

In this paper, we hypothesize that foot-based audits are theoretically likely to represent the most accurate method of capturing fine detail local environment features required for health behaviour studies whilst acknowledging that this may not act as a ‘gold standard’ method (Gasevic et al., 2011). We have therefore examined criterion validity (e.g. the level of agreement between the physical (criterion) and virtual (test) measures) using the ICC as done in previous studies (Badland et al., 2010). We report Pearson correlation coefficients for continuous variables in Appendix S2. With a real ‘gold standard’ the Pearson correlation coefficient provides an indication of the direct relationship with power loss, efficiency and bias from using the error-prone (test) measure (Checkoway et al., 2004). A walkability index was constructed for both the foot-based and GSV audits using latent class analysis (LCA). The following 10 built environment variables were used: pavement quality, lowered curbs, barriers on pavement, pavement width, pedestrian traffic, road use, road connectivity, traffic calming measures, lamp posts and road crossings (see Appendix S3 and Hawkesworth et al., 2015). For all variables, the comparison of the GSV audits with foot-based audits was conducted at the segment level. Correlations and agreement could not be computed for health promotion adverts because there was no variation in GSV ratings.

Segment level data from foot-based audits were aggregated at the LSOA level to allow analysis and comparison to secondary data. Comparison of data collected by secondary data sources and foot-based audits was assessed using the ICC from one-way random effects models using the cut-offs described above. We attempted to find and best define routinely available secondary data that reflected data captured by the foot-base audits. By definition, it was not possible to always compare like with like but we aimed to assess how well different built environment descriptors were correlated from the different data sources. Total traffic in foot-based audits was counted for a 5 min interval during each segment audit and was estimated for 1 h in order to provide a more consistent scale when comparing traffic volume between the foot-based audits and secondary data. The predominant land use category was used to estimate the proportion of segments that were open green areas and to generate an entropy score at LSOA level in the foot-based audits. For this paper, the dataset of 17 English towns was used to compare selected environmental variables between primary and secondary data sources at the LSOA level because some secondary neighbourhood level data is collected differently in Scotland compared to England. All analyses were conducted in Stata 14.1 (StataCorp LP, 2015).

## 3 Results

Summary statistics for selected environmental variables are presented in Table 1. Complete descriptions for the remaining environmental variables are available in Appendix S4. Environmental data were collected for 820 LSOAs, where participants of the BRHS and BWHHS cohort studies live, in 17 English towns through foot-based audits and secondary data sources. A total of 1,396 segments covering 87 LSOAs in two English towns (Ipswich and Newcastle-under-Lyme) were audited through foot based and GSV audits.

Below we report on the IRR of foot-based and GSV audit tools and comparisons between foot-based audits, GSV audits and routinely collected secondary data sources.

### 3.1 Inter-rater reliability of foot-based audits

The analysis of IRR for the foot-based audits included 174 repeat segments in 29 LSOAs in the study pilot towns of Guildford and Bristol. Five different fieldworkers, working in rotating pairs, conducted repeat segments as part of the reliability study. Overall there was extremely good agreement between different foot-based auditors on all the variables included on the audit tool (Table 2). Some of the more subjective variables such as the quality of pavements or the adequacy of lowered curbs showed less agreement than more objective variables such as road connectivity. Counts of amenities, shops and services showed excellent agreement. The poorest agreement was shown for counts of health promotion adverts, which were rarely seen (Table 1, Table 2). In contrast, counts of adverts for unhealthy products (food, alcohol or cigarettes) showed excellent agreement between observers (Table 2).

### 3.2 Inter-rater reliability of GSV audits

The analysis of IRR for the GSV audits included 104 randomly selected segments (13% of total segments) in 43 LSOAs in Ipswich. Two fieldworkers collected repeated segment information as part of the reliability study. The IRR for comparing use of the GSV audit tool showed similar results to that of the foot based audits (Table 2). More objective items, such as transport-related items and counts of shops and services, had a better agreement than more subjective items (ie. items required judgement by the observer), such as availability of green space. Unsurprisingly, in the GSV audits, agreement between fieldworkers for counts of adverts and for aesthetic measures was fair or poor, possibly due to the limited ability to view such items through GSV. Similarly, for built environment items better agreement was observed in more objective road network related items (e.g. connectivity) but agreement was lower for more subjective items, or finer detail items, such as lowered curbs and slope that may only be easily assessed by audits done on the ground. Land use items showed moderate to substantial agreement (Table 2).

### 3.3 Comparison of foot-based and GSV audit tools

The analysis of agreement showed that transport-related items (e.g. road crossings and bus stops) and counts of shops and services had the highest ICC values (Table 3). More specifically, ICC was 0.92 (95% CI 0.92–0.93) for bus stops, 0.76 (95% CI 0.74–0.78) for road crossing and 0.88 (0.87–0.89) for shops and services. Lower agreements were observed for the majority of the more subjective items or those items that are harder to measure in GSV, such as access to green space (ICC=0.33, 95% CI 0.29–0.38) and footpaths (ICC=0.37, 95% CI 0.32–0.41), counts of adverts (ICC=0.18, 95% CI 0.13–0.24), built environment items (except road connectivity; kappa=0.80, 95% CI 0.77–0.83) and aesthetics, particularly security measures (kappa =0.19, 95% CI 0.15–0.25) and graffiti (kappa =0.04, 95% CI 0.00–0.08). From the land use field, predominant land use showed substantial agreement (kappa =0.63, 95% CI 0.59–0.67) whereas secondary land use showed fair agreement (kappa=0.33, 95% CI 0.29–0.38). The class-specific latent class indicator probabilities for the foot-based and GSV LCAs for the walkability index are given in Appendix S3. The characteristics of the latent classes in the foot-based audit and GSV LCAs were very similar, with class 1 in the foot-based audit LCA equivalent to class 2 in the GSV LCA; class 2 in the foot-based audit LCA equivalent to class 3 in the GSV LCA; and class 3 in the foot-based audit LCA equivalent to class 1 in the GSV LCA. The 3-class model was therefore chosen as most appropriate in both GSV and foot-based LCAs. The poor agreement in the walkability index may reflect the fair to moderate agreement observed for the built environment variables that were used in the LCA to construct the index (Table 3).

### 3.4 Comparison of foot-based audit with routinely available secondary data

We compared nine variables related to transport, services and land use between our foot-based audit data and secondary data measures. Overall, there was fair or good agreement for items that we were able to directly compare (Table 4). This included counts of bus stops (ICC=0.56, 95% CI 0.51–0.60) and medical services (ICC=0.79, 95% CI 0.77–0.82) that were measured using very similar approaches in the two methods. However, variables for traffic volume, proportion of green space and land use mix were generated or available in ways that may not be directly comparable between the two methods and produced very poor agreement (Table 4).

### 3.5 Resources required: comparison of time and estimated costs in collecting foot-based audit, GSV audit and routinely available secondary data

We estimated that foot-based audits took, on average, 20 days per town to complete and an additional five days per town were spent for data entry. GSV audits took, on average, 12 days per town to complete. Time taken per audited segment was considerably higher in foot-based compared to GSV audits (9.5 vs 6.4 min per segment). Foot-based fieldworkers audited, on average, 15 segments per pair/day whereas the GSV fieldworkers audited 55 segments per person/day. Staff travel time and expenses to audit areas meant considerably higher costs for foot-based audits. Foot-based audits in the two towns were done in approximately the same season and took the same time to complete. Overall, there were no quality issues with GSV imagery. The identification, use and processing of secondary data sources required considerably less time and covered 820 LSOAs across the 17 English towns. However, only a small proportion of audited items were generated using these freely available secondary data sources as only a small number of variables were comparable between foot-based and these sources (Table 5).

## 4 Discussion

### 4.1 Main findings

To our knowledge this is the first study to compare three different methods of objectively measuring features of the built environment used to examine the association between neighbourhood and health. This study demonstrates a good agreement between foot-based and GSV audit tools used to collect primary data on a range of built environment domains, but highlights the fact that secondary and primary data sources are often measuring very different aspects of the environment. Both direct observations (either on foot or virtually through GSV) and routinely available secondary data sources have a role to play in characterising the environment in studies of the impact of the built environment and health (Fleischhacker et al., 2013, Krenn et al., 2011, Schaefer-McDaniel et al., 2010) but this study highlights the importance of understanding how well the approaches assess the different environmental domains. Secondary data are often used as a proxy measure of aspects of the environment such as the ‘walkability’ of an area assessed by street connectivity and residential density (Leslie et al., 2007). In contrast, direct observations can provide much more detailed and context relevant environmental data required to understand the complexity of the associations between people and place (Feng et al., 2010, Sallis et al., 2006, Van Cauwenberg et al., 2011).

The results of the IRR analysis demonstrated higher values in the foot-based compared to the GSV audits. This may be partly explained by the larger variation between segments in foot-based audits, and IRR analysis undertaken in two towns compared to one town for GSV audits. The comparison of GSV audit against foot-based audit demonstrated a good to excellent or substantial agreement for almost all of the more objective items of the tool. These include transport-related items, shops and services, and the road connectivity item from the built environment section. There was less concordance for more subjective assessments, such as pavement quality. Our study findings are consistent with findings from a recent systematic review on using remote geospatial tools to characterise the neighbourhood environment (Charreire et al., 2014). Virtual street audits are promoted as a resource-efficient alternative to foot-based assessments (Fleischhacker et al., 2013, Wilson et al., 2012) and the results of our study mostly confirm this, although some of the difference in the time taken per audited segment between the foot-based and GSV audits may be explained by the absence of data collection for shop prices and traffic volume during the GSV audit. Other studies have also shown that GSV audits are less time consuming (Bethlehem et al., 2014, Charreire et al., 2014) or found no time difference (Wu et al., 2014).

Comparisons of secondary data sources to foot-based audit data demonstrated mixed results in this study. There was fair to excellent agreement for items that were measured using similar scales (e.g. counts of bus stops). The use of different methodologies or scales used to collect data may explain the lack of agreement or presence of negative ICC scores for some items (e.g. traffic volume). Such negative ICC values are not theoretically possible but could be observed in estimates when the observed variation between units (ie. LSOAs) is even less than you would expect given the differences within LSOAs (Giraudeau, 1996).

We found that identifying and processing secondary data sources that are publicly accessible were less time consuming than foot-based and GSV audits, but these could only be used for a very limited number of environmental measures we wanted to study. Previous built environment studies comparing secondary sources with foot-based audits were usually undertaken to assess the validity and/or reliability of secondary data sources (Evenson and Wen, 2013, Fleischhacker et al., 2013). We could have used other secondary data sources, however use of such sources usually involves requesting data from local government (Burgoine and Harrison, 2013, Cummins and Macintyre, 2009, Lake et al., 2012), licence agreements (Burgoine and Harrison, 2013) or commercial business listing companies (Evenson and Wen, 2013, Lake et al., 2012, Liese et al., 2013, Paquet et al., 2008). Such approaches require additional, sometimes considerable, time and financial resources for data acquisition, data cleaning and processing (Evenson and Wen, 2013).

### 4.2 Methodological issues

A number of methodological issues arise when considering use of foot-based and/or virtual street audits and secondary data sources including the quality and completeness of data collection, data aggregation, documentation and management, auditing times and research costs, and temporality (Brownson et al., 2009, Charreire et al., 2014). Selection of one data collection method over another should depend on the needs of the study, the research questions and costs and benefits related to the use of each method (Bader et al., 2010, Curtis et al., 2013, Gravlee et al., 2006, Shareck et al., 2012). Foot-based audits require training and monitoring of staff to ensure data quality both during collection and data entry (Brownson et al., 2009). The same applies for virtual street audits in GSV. High ICC scores for an item require adequate operational definition and proficient observers (Shareck et al., 2012, Zenk et al., 2007). Good data management protocols may reduce the risk of missing data in foot-based audits. However, data completeness and quality is a known limitation for GSV as coverage may not be geographically complete, images may be obscured (Charreire et al., 2014) and visual inspection of the area is limited to images provided on a particular date, and therefore out of the control of observers. Data documentation and detailed item definitions in a tool is important to enable replication, as there is heterogeneity in defining environmental items between tools (Brownson et al., 2009, Charreire et al., 2014). GIS-based measures produced from secondary data sources may require substantial time to clean, manage and analyze especially if data come from less routinely collected sources (Brownson et al., 2009). An important consideration is validity of secondary data sources as inaccuracies in these data may obscure relationship between neighbourhood and health (Cummins and Macintyre, 2009). Equally important, the use of different spatial scales to aggregate segment-level data may make comparisons of secondary data sources with foot-based audits and comparison with other studies problematic, as neighbourhood definition may vary considerably. This also raises the issue of measurement error, scale and specificity of environmental items included in the audit tool (Brownson et al., 2009, Fleischhacker et al., 2013, Pliakas et al., 2014, Shareck et al., 2012). For example, we were able to directly compare variables expressed as counts on a continuous scale (e.g. count of bus stops and shops) when these were available in this format in both the foot-based audits and in secondary data sources. However, categorical variables in foot-based audits (eg. land use variables) were converted to a proportion of segments having a specific characteristic (ie. green space) within a LSOA. This measure depends on how segments were defined, number of segments audited in the LSOA and the area of the LSOA. It is therefore likely to differ from the equivalent measure from secondary data sources, where land use items are usually expressed as a percentage of the area of the LSOA, even if each segment measure is accurate. Thus we could not directly compare secondary data sources to foot based audits for some items. Finally, the use of a tool designed to concurrently measure a wide range of environmental dimensions relevant to multiple health outcomes, for example studies attempting to capture the ‘obesogenic environment’, may have limited utility if information is missing on specific features of the environment that are theoretically linked to specific health outcomes (Shareck et al., 2012).

To increase accuracy of reporting, and decrease problems with matching segments between foot-based and GSV audits it is crucial to identify discrepancies in the start and end points of the segments (eg. road names recorded on the ground may be different to the way roads are recorded in GSV). An important consideration for GSV audits is temporality due to the date imagery was uploaded, which might cause problems for some of the items audited (Charreire et al., 2014). In our study, one study town (Ipswich) had a very small time gap between GSV and foot-based audits (7 months) but the gap was much longer, between 1 and 4 years, for the other study town (Newcastle-under-Lyme). Daily variation, for example in pedestrian traffic or adverts, may also explain low agreement scores between the field-based and GSV audits (Charreire et al., 2014, Wu et al., 2014). Subjective items have been found to be consistently less reliable than more objective items (Wilson et al., 2012), however, another plausible explanation is that such items may be easily missed in virtual audits due to insufficient resolution in GSV (Wu et al., 2014). This was particularly difficult when trying to distinguish the different type of shops or identifying parking restrictions.

### 4.3 Future research directions

Our paper contributes to the development of methods for measuring elements of the built environment in future public health research. Such studies using a wide range of data sources and methods to assess links between neighbourhood environments and multiple health behaviours will help develop tools that include more refined measurement items and inform how best to allocate research resources (Brownson et al., 2009).

This study sets out some of the advantages (and disadvantages) for using a single primary data collection tool that can be used for both foot based and GSV audits across multiple environmental domains. A single data collection audit tool may not always be the most useful or feasible method of capturing multiple potential exposures operating at different spatial scales. Future built environment research should consider the need to design studies *a priori* using multiple methodological approaches and data sources to capture features that operate on health behaviours at different spatial scales. Engagement and participation of people living or using a neighbourhood should be sought as this is important in the development, or adaptation, of audit tools with the inclusion of items that may be particularly relevant for specific population groups or contexts (Brownson et al., 2009). Studies of community perceptions of environmental features, or of public-environment interactions such as mapping activity spaces for different population groups (Milton et al., 2015) should also be included where possible to capture, and contrast, different influences on health behaviours.

Capacity building is an important component of built environment research as data collection through observation or virtual audits requires considerable investment in staff time and training (Brownson et al., 2009). Recruiting fieldworkers locally can achieve cost savings. Familiarity of the use of GSV is essential to reduce time taken to undertake audits remotely (Badland et al., 2010). As foot-based audits may be superior to other methods when collecting fine detail environmental features, such as path quality or in-store measures, the use of mobile and web applications, tablets, and smartphones that are linked to a central repository or database can be used to reduce time for data collection and entry substantially (Aanensen et al., 2009, Brownson et al., 2009, Gravlee et al., 2006). However, the use of this technology should be weighed against the cost of purchasing such equipment and software and their feasibility in the field should be tested, especially in low income settings or areas of high crime where they may pose a safety risk to fieldworkers (Brownson et al., 2009, Charreire et al., 2014). Remote audits may be a good solution where safety is of concern.

Collaboration with local governmental departments, including public health, planning, and transport, may provide valuable sources of routine secondary data that are not usually used (Brownson et al., 2009) although additional resources may be required to process the data for research use as it will be collected in differing ways. Improving the availability of spatial data from governmental departments (eg. licensed premises data) (Local Government Association, 2013) in a usable format would greatly reduce costs and time. More recently, crowdsourcing has been combined with GSV to assess street-level characteristics of the built environment (Hara et al., 2013, Hara et al., 2014). Although not yet available, GSV may in the future provide the ability to view multiple dates of imagery in places that could offer the potential to remotely assess environmental change longitudinally at a finer spatial scale. This could present an opportunity to evaluate complex natural experiments such as area regeneration or urban design interventions (Charreire et al., 2014, Wilson et al., 2012). Finally, the use of GSV audit tools may be particularly valuable in large national or international projects which include multiple study towns or countries in order to assess diverse environments cost- effectively (Badland et al., 2010). However, ethical considerations should be addressed adequately in order to protect individuals’ privacy as researchers are increasingly raising questions about the use of online tools, such as GSV, that indirectly identify where study participants live.

## 5 Conclusion

There is increasing interest in the potential role of the built environment in influencing health behaviours such as physical activity but understanding these complex relationships requires accurate methods for objectively measuring relevant environmental features. Our study again highlights the fact that primary and secondary data sources often measure very different aspects of the environment. There is often ambiguity in the literature about what secondary data can usefully capture in studies addressing the effects of place on health and this comparison helps to highlight the different hypotheses and scales that can be studied by these complimentary methodologies. GSV is a reliable tool, particularly for more objective physical measures of the built environment. We found foot-based audits are superior when collecting data on fine detail environmental features. The use of secondary data sources can be used to derive a range of environmental measures, if validated properly, and are useful when collected on a routine basis to avoid any temporality issues. Studies of the association between the built environment and health or health behaviours need to ensure that they are employing the most relevant and optimal methods for their research question whether this be direct observation, secondary data or surveys of individuals’ neighbourhood perceptions. A single method may not always be the best approach for capturing multiple potential exposures operating at different spatial scales in complex health systems, such as ‘obesogenic environments’. Future built environment research should consider the need to design studies *a priori* using multiple approaches and varied data sources in order to capture features that operate on health behaviours at different scales to provide a more complete understanding of the influence of the local environment on health.

## Contributorship statement

KL, BA, JPC, CG and RM conceived the study. KL and SH designed and managed the environmental data collection with input from CG, KN and TP. TP generated the environmental variables from secondary data sources. TP conducted the data analysis with input from BA and RS. TP wrote the first draft of the manuscript with KL, SH and PW. JPC is Co-Director of the British Women's Heart and Health Study. RM is Co-Investigator of the British Regional Heart Study. All authors contributed to the scientific content of the manuscript and approved the final version for publication.

## Figures and Tables

**Table 1:** Summary statistics for selected built environment variables collected through foot-based audits, Google Street View audits and secondary data.

**Variable**	**Foot-based audits**				**Google Street View audits**				**Secondary data**			
	**Mean (or n)**	**SD (or %)**	**Min**	**Max**	**Mean (or n)**	**SD (or %)**	**Min**	**Max**	**Mean**	**SD**	**Min**	**Max**
**LSOA level (n=820, unless otherwise stated)**												

*Transport*												
Traffic volume (n=252^a^)												
Counts from FBA aggregated at LSOAa1	5188	4685.1	0	34392								
Segment from FBA with maximum value at LSOA levela2	1000	675.4	0	3876								
AADF aggregated at LSOAa1									18402	15802.2	93	93303
Road traffic injuries									432	390.4	0	2866
Bus stops^b^	7	5.17	0	40					10	6.68	0	57
Proportion of roads classified as motorways or A-roads^c^	0.18	0.133	0	1					0.05	0.090	0	0.60

*Services*												
Pharmacies	0.3	0.76	0	11					0.3	0.68	0	6
GPs	0.2	0.47	0	3					0.3	0.69	0	8
Dentists	0.3	0.91	0	11					0.2	0.86	0	11
Medical services (Pharmacies, GPs, and dentists)	0.7	1.65	0	23					0.8	1.79	0	23

*Availability of green space and land use*												
Proportion of green space^d^	6.9	10.01	1.00	75.00					65.7	16.90	2.59	97.99
Land use mix^e^	0.5	0.22	0.10	0.98					0.6	0.19	0.08	0.96

**Segment level (n=1396)**												

*Transport*												
Road Crossings^f^	0.3	1.04	0	11	0.3	0.95	0	11				
Bus stops	0.5	1.21	0	10	0.4	1.16	0	10				
*Amenities*^g^	0.6	1.99	0	37	0.8	2.13	0	37				

*Availability of green space and footpaths*												
Availability of green space^h^	0.1	0.40	0	4	0.2	0.48	0	5				
Trails and footpaths^i^	0.6	1.09	0	11	0.6	1.12	0	21				
*Shops and services*^j^	1.1	4.33	0	100	0.9	3.58	0	74				

*Adverts*												
Health promotion adverts^k^	0.0^m^	0.21	0	4	0.0^n^	0.06	0	1				
Unhealthy product promotion^l^	0.2	1.21	0	20	0.0^o^	0.44	0	13				

AADF: Annual average daily flow for all motor vehicles; GP: General Practitioner; LSOA: Lower layer super output area; SD: Standard deviation.

a In the foot based audit, total traffic volume was estimated as the number of total traffic for 1 h. In the secondary data, total traffic volume is expressed as vehicles per day.

a1 Number of motorized vehicles and buses, from foot based audits, and AADF, from secondary data, aggregated at the LSOA level.

a2 Using the foot-based audited segment with the maximum value of count for each LSOA.

b Bus stops with and without shelter.

c Estimated proportion of segments in the foot based audits with 3+ lanes and 2-way with lane marks road types (Segments classified as N/A were excluded).

d Estimated proportion of segments in the foot based audits where the predominant land use was classified as open green areas.

e Spatial entropy score. Residential land use included purpose built block of flats, offices / shops with flats above, terraced houses and detached or semi-detached houses. Non-residential land use included offices, shops and services, schools, industrial / other commercial buildings / car parks. Other land type included derelict or vacant building/plot and n/a. Green space land type included open green areas.

f Road crossings: Number of traffic lights with and without pedestrian indicators, Zebra or Pelican crossings, lowered curbs or traffic islands and under passes, over passes or bridges;

g Amenities: Number of benches, public toilets, post and phone boxes, public and commercial bins and recycling locations;

h Availability of green space: Number of small green/paved areas and access points to large parks;

i Trails and footpaths: Number of walking trails and alleys/connecting paths;

j Shops and services: Number of independent convenience stores, small supermarkets, large supermarkets with parking, off-licence, fast food outlet, restaurants, other food shops, cafes, pubs, hotels, non-food shops, pharmacy, GP, NHS and private dentists, hospitals, other healthcare, residential homes, religious centres, private nursery school, leisure centres, public swimming pools, laundrettes and hairdressers, banks and post offices, recreational venues, shopping centres.

k Health promotion adverts encountered on segment (from shops, billboards or other sources) including smoking cessation, commercial healthy food, non-commercial food, commercial and non-commercial physical activity.

l Unhealthy product promotion encountered on segment (from shops, billboards or other sources) including alcoholic drinks, sugary drinks and unhealthy/snack/junk food promotions.

m Mean is 0.023.

n Mean is 0.004.

o Mean is 0.039.

**Table 2:** Inter-rater reliability using Kappa and Intraclass Correlation Coefficients.^a^

**Item**	**Foot-based audits (174, unless otherwise stated)**			**Google Street View audits (n=104)**		
	**Individual ICC**	**95% CIs**	**Agreement**	**Individual ICC**	**95% CIs**	**Agreement**
*Transport*						
Traffic volume^b^	0.99	0.99–1.0	Excellent	N/A	N/A	N/A
Road Crossings	0.94	0.92–0.96	Excellent	0.79	0.71–0.86	Excellent
Bus stops	0.88	0.84–0.91	Excellent	0.88	0.82–0.91	Excellent
*Amenities*	0.99	0.99–0.99	Excellent	0.66	0.53–0.75	Good

*Availability of green space and footpaths*						
*Availability of* green space	0.97	0.96–0.98	Excellent	0.56	0.41–0.68	Fair
Trails and footpaths (n=173)	0.92	0.89–0.94	Excellent	0.48	0.31–0.61	Fair
*Shops and services*	0.99	0.98–1.0	Excellent	0.81	0.74–0.87	Excellent

*Adverts*						
Health promotion	0.58	0.47 −0.67	Fair	-c	-c	-c
Unhealthy products	0.94	0.92–0.95	Excellent	0.18	0.13–0.24	Poor
	**Kappa**	**95% CIs**	**Agreement**	**Kappa**	**95% CIs**	**Agreement**

*Built environment*						
Pavement quality (n=173)	0.70	0.63–0.81	Substantial	0.45	0.28–0.57	Moderate
Lowered curbs (n=169)	0.68	0.54–0.79	Substantial	0.36	0.21–0.47	Fair
Barriers on pavement (n=170)	0.66	0.52–0.83	Substantial	0.43	0.24–0.62	Moderate
Pavement width (left)	0.71	0.62–0.79	Substantial	0.48	0.26–0.59	Moderate
Pavement width (right) (n=173)	0.69	0.58–0.76	Substantial	0.53	0.37–0.64	Moderate
Pedestrian traffic (n=173)	0.68	0.59–0.78	Substantial	0.35	0.13–0.50	Fair
Road use (n=173)	0.85	0.76–0.93	Almost perfect	0.70	0.51–0.87	Substantial
Road connectivity (n=172)	0.92	0.82–0.97	Almost perfect	0.77	0.60–0.89	Substantial
Traffic calming (n=173)	0.81	0.72–0.92	Almost perfect	0.67	0.40–0.91	Substantial
Parking spaces	0.88	0.80–0.94	Almost perfect	0.57	0.43–0.67	Moderate
Parked cars (n=172)	0.75	0.66–0.81	Substantial	0.33	0.19–0.56	Fair
Lamp posts (n=170)	0.69	0.57–0.77	Substantial	0.49	0.36–0.65	Moderate
Slope (n=173)	0.84	0.78–0.91	Almost perfect	0.31	0.18–0.50	Fair

*Aesthetics*						
Neighbourhood watch (n=167)	0.80	0.70–0.88	Almost perfect	0.29	0.16–0.42	Fair
Security measures (n=171)	0.89	0.79–0.97	Almost perfect	0.03	−0.04–0.15	Poor
Greenery (n=172)	0.85	0.79–0.91	Almost perfect	0.22	0.12–0.31	Fair
Graffiti (n=171)	0.96	0.86–1.00	Almost perfect	−0.01	−0.03–0.00	Poor
Litter (n=173)	0.56	0.42–0.74	Moderate	N/A	N/A	N/A

*Land use*						
Predominant land use (n=171)	0.77	0.66–0.87	Substantial	0.62	0.34–0.77	Substantial
Secondary land use (n=76)	0.76	0.60–0.87	Substantial	0.48	0.34–0.63	Moderate

CIs: Confidence Intervals; ICC: Intraclass Correlation Coefficient; N/A: Data were not collected for this item.

a Cut-off values for kappa statistic: 0.80–1.00 (almost perfect), 0.60–0.79 (substantial), 0.40–0.59 (moderate), 0.20–0.39 (fair) and 0.00–0.19 (poor). Cut-off values for ICC: 0.75–1.00 (excellent), 0.60–0.74 (good), 0.40–0.59 (fair) and 0.00–0.39 (poor).

b Total traffic volume counted, in the original scale, for 5 min interval during segment audit.

c There was no variation in Google Street View ratings.

**Table 3:** Comparison of the foot-based audits vs Google Street View audits at segment level using Kappa and Intraclass Correlation Coefficients (n=1,396).^a^

**Variable**	**Individual ICC**	**95% CIs**	**Agreement**
*Transport*			
Road Crossings	0.76	0.74 – 0.78	Excellent
Bus stops	0.92	0.92 – 0.93	Excellent
*Amenities*	0.66	0.63 – 0.69	Good

*Availability of green space and footpaths*			
*Availability of* green space	0.33	0.29 – 0.38	Poor
Trails and footpaths	0.37	0.32 – 0.41	Poor
*Shops and services*	0.88	0.87 – 0.89	Excellent

*Adverts*			
Health promotion	-b	-b	-b
Unhealthy product promotion	0.18	0.13 – 0.24	Poor
	**Kappa**	**95% CIs**	**Agreement**

Walkability index (n=1,271)	0.05	0.01 – 0.12	Poor

*Built environment*			
Pavement quality (n=1,377)	0.36	0.32 – 0.40	Fair
Lowered curbs (n=1,333)	0.41	0.38 – 0.47	Moderate
Barriers on pavement (n=1,367)	0.35	0.28 – 0.39	Fair
Pavement width (left) (n=1,358)	0.54	0.48 – 0.57	Moderate
Pavement width (right) (n=1,353)	0.51	0.47 – 0.54	Moderate
Pedestrian traffic (n=1,371)	0.21	0.16 – 0.25	Fair
Road use (n=1,376)	0.49	0.44 – 0.54	Moderate
Road connectivity (n=1,388)	0.80	0.77 – 0.83	Substantial
Traffic calming (n=1,371)	0.53	0.48 – 0.61	Moderate
Parking spaces (n=1,378)	0.50	0.46 – 0.54	Moderate
Parked cars (n=1,385)	0.39	0.34 – 0.42	Fair
Lamp posts (n=1,384)	0.40	0.36 – 0.45	Moderate
Slope (n=1,382)	0.44	0.39 – 0.48	Moderate

*Aesthetics*			
Neighbourhood watch (n=1,375)	0.28	0.25 – 0.32	Fair
Security measures (n=1,375)	0.19	0.15 – 0.25	Poor
Greenery (n=1,377)	0.22	0.17 – 0.27	Fair
Graffiti (n=1,373)	0.04	0.00 – 0.08	Poor

*Land use*			
Predominant land use (n=1,351)	0.63	0.59 – 0.67	Substantial
Secondary land use (n=823)	0.33	0.29 – 0.38	Fair

CIs: Confidence Intervals; ICC: Intraclass Correlation Coefficient.

a Cut-off values for kappa statistic: 0.80–1.00 (almost perfect), 0.60–0.79 (substantial), 0.40–0.59 (moderate), 0.20–0.39 (fair) and 0.00–0.19 (poor) [6]. Cut-off values for ICC: 0.75–1.00 (excellent), 0.60–0.74 (good), 0.40–0.59 (fair) and 0.00–0.39 (poor).

b There was no variation in Street View ratings.

**Table 4:** Comparison of the foot-based audits vs secondary data at LSOA level using Intraclass Correlation Coefficients (n=820, unless otherwise stated).^a^.

**Variable**	**Individual ICC**	**95% CIs**	**Agreement**
*Transport*			
Traffic volume using AADF (n=252^b^)	−0.39	−0.49 −0.28	Poor
Traffic volume using RTIs	−0.38	−0.44 −0.32	Poor
Bus stops	0.56	0.51–0.60	Fair
Proportion of roads classified as motorways or A-roads	−0.11	−0.18 −0.04	Poor

*Services*			
Pharmacies	0.65	0.61–0.69	Good
GPs	0.42	0.36–0.47	Fair
Dentists	0.71	0.67–0.74	Good
Medical services (GPs, dentists, pharmacists)	0.79	0.77–0.82	Excellent

*Availability of green space*			
Proportion of greenspace	−0.75	−0.78 to −0.72	Poor

*Land use mix*			
Spatial entropy score	−0.29	−0.35 to −0.23	Poor

AADF: Annual average daily flow for all motor vehicles; CIs: Confidence Intervals; FBA: Foot based audits; GP: General Practitioner; ICC: Intraclass Correlation Coefficient; RTIs: Road traffic injuries.

a Cut-off values for kappa statistic: 0.80–1.00 (almost perfect), 0.60–0.79 (substantial), 0.40–0.59 (moderate), 0.20–0.39 (fair) and 0.00–0.19 (poor) [6]. Cut-off values for ICC: 0.75–1.00 (excellent), 0.60–0.74 (good), 0.40–0.59 (fair) and 0.00–0.39 (poor).

b Only LSOAs with at least one count point that links the AADFs to the road network.

**Table 5:** Comparison of time and potential costs for managing, collecting and cleaning data using the three methods to objectively assess the neighbourhood environment.

	**Foot-based audits**		**Google Street View audits**		**Secondary data**^a^
	**Newcastle-under-Lyme**	**Ipswich**	**Newcastle-under-Lyme**	**Ipswich**	
**Data collection**					
Period	Nov 2013 - Dec 2013	Oct 2011 - Dec 2011	May 2014 - Aug 2014	Dec 2013 - May 2014	May−15
Dates most GSV images were uploaded	N/A	N/A	Mar 2009 and Nov 2012	Jul 2012	N/A
N of staff^b^	4	4	1	1	1
N of segments audited^d^	588	626	653	716	N/A
N of days	22	18	13^e^	12^e^	10^c^
Minutes per segment^d^	8.9	10.1	7	5.8	N/A
Transport	1 h/person	1 h/person	N/A	N/A	N/A

**Data entry**					
N of staff	1	1	N/A	N/A	N/A
N of hours	30	32	N/A	N/A	N/A

GSV: Google Street View; N: Number; N/A: Not applicable.

a Secondary data for all 17 English towns.

b Staff are fieldworkers for FBA audits and GSV audit in Newcastle and data analyst for GSV audit in Ipswich and secondary data.

c Time includes identifying potential data sources and processing data in MS Excel, ArcGIS and STATA for land-use, traffic volume, public transport and selected services.

d 1,369 segments in GSV audits and 1,214 segments in foot-based audits with valid start and end times.

e Full-time equivalent.

## References

[bib1] Aanensen D.M., Huntley D.M., Feil E.J., al-Own Fa, Spratt B.G. (2009). EpiCollect: linking smartphones to web applications for epidemiology, ecology and community data collection. PLoS One.

[bib2] Armstrong B.K., White E., Saracci R. (1992). Principles of Exposure Measurement in Epidemiology.

[bib3] Bader M.D., Ailshire J.A., Morenoff J.D., House J.S. (2010). Measurement of the local food environment: a comparison of existing data sources. Am. J. Epidemiol..

[bib4] Badland H.M., Opit S., Witten K., Kearns R.A., Mavoa S. (2010). Can virtual streetscape audits reliably replace physical streetscape audits?. J. Urban Health.

[bib5] Ben-Joseph E., Lee J.S., Cromley E.K., Laden F., Troped P.J. (2013). Virtual and actual: relative accuracy of on-site and web-based instruments in auditing the environment for physical activity. Health Place..

[bib6] Bethlehem J.R., Mackenbach J.D., Ben-Rebah M., Compernolle S., Glonti K., Bardos H., Rutter H.R., Charreire H., Oppert J.M., Brug J., Lakerveld J. (2014). The SPOTLIGHT virtual audit tool: a valid and reliable tool to assess obesogenic characteristics of the built environment. Int J. Health Geogr..

[bib7] Brownson R.C., Hoehner C.M., Day K., Forsyth A., Sallis J.F. (2009). Measuring the built environment for physical activity: state of the science. Am. J. Prev. Med..

[bib8] Burgoine T., Alvanides S., Lake A. (2013). Creating 'obesogenic realities'; do our methodological choices make a difference when measuring the food environment?. Int J. Health Geogr..

[bib9] Burgoine T., Harrison F. (2013). Comparing the accuracy of two secondary food environment data sources in the UK across socio-economic and urban/rural divides. Int J. Health Geogr..

[bib10] Calogiuri G., Chroni S. (2014). The impact of the natural environment on the promotion of active living: an integrative systematic review. BMC Public Health.

[bib11] Caspi C.E., Sorensen G., Subramanian S.V., Kawachi I. (2012). The local food environment and diet: a systematic review. Health Place..

[bib12] Charreire H., Casey R., Salze P., Simon C., Chaix B., Banos A., Badariotti D., Weber C., Oppert J.M. (2010). Measuring the food environment using geographical information systems: a methodological review. Public Health Nutr..

[bib13] Charreire H., Mackenbach J.D., Ouasti M., Lakerveld J., Compernolle S., Ben-Rebah M., McKee M., Brug J., Rutter H., Oppert J.M. (2014). Using remote sensing to define environmental characteristics related to physical activity and dietary behaviours: a systematic review (the SPOTLIGHT project). Health Place..

[bib14] Checkoway H., Pearce N., Kriebel D. (2004). Research Methods in Occupational Epidemiology.

[bib15] Christian H., Zubrick S.R., Foster S., Giles-Corti B., Bull F., Wood L., Knuiman M., Brinkman S., Houghton S., Boruff B. (2015). The influence of the neighborhood physical environment on early child health and development: a review and call for research. Health Place..

[bib16] Cicchetti D. (1994). Guidelines, criteria and rules of thumb for evaluating normed and standardized assessment instruments in psychology. Psychol. Assess..

[bib17] Clarke P., Ailshire J., Melendez R., Bader M., Morenoff J. (2010). Using Google Earth to conduct a neighborhood audit: reliability of a virtual audit instrument. Health Place..

[bib18] Cummins S., Curtis S., Diez-Roux A.V., Macintyre S. (2007). Understanding and representing 'place' in health research: a relational approach. Soc. Sci. Med..

[bib19] Cummins S., Macintyre S. (2009). Are secondary data sources on the neighbourhood food environment accurate? Case-study in Glasgow, UK. Prev. Med..

[bib20] Cummins S., Macintyre S., Davidson S., Ellaway A. (2005). Measuring neighbourhood social and material context: generation and interpretation of ecological data from routine and non-routine sources. Health Place..

[bib21] Curtis J.W., Curtis A., Mapes J., Szell A.B., Cinderich A. (2013). Using google street view for systematic observation of the built environment: analysis of spatio-temporal instability of imagery dates. Int J. Health Geogr..

[bib22] de Vet E., de Ridder D.T., de Wit J.B. (2011). Environmental correlates of physical activity and dietary behaviours among young people: a systematic review of reviews. Obes. Rev..

[bib23] Department for Communities and Local Government, 2013. Index of Multiple Deprivation 2010, Local Authority District Rank of Average Rank. Available at: 〈http://opendatacommunities.org/data/societal-wellbeing/deprivation/imd-rank-la-2010〉 (accessed 27.01.16). Archived at: 〈http://www.webcitation.org/6eqnNh0Qm〉 on 27 January 2016.

[bib24] Department for Transport, 2014. National Public Transport Access Node. Available at: 〈https://www.gov.uk/government/publications/national-public-transport-access-node-schema〉 (accessed 27.01.16). Archived at: 〈http://www.webcitation.org/6eqnduU44〉 on 27 January 2016.

[bib25] Department for Transport, 2015. Annual Average Daily Traffic Flows. Available at: 〈http://www.dft.gov.uk/traffic-counts/〉 (accessed 27.01.16). Archived at: 〈http://www.webcitation.org/6eqo6hpmM〉 on 27 January 2016.

[bib26] Digimap Ordnance Survey, 2015. Meridian 2 National. Available at: 〈http://digimap.edina.ac.uk/webhelp/os/data_information/os_products/meridian_2.htm〉 (accessed 27.01.16). Archived at: 〈http://www.webcitation.org/6eqo18lK6〉 on 27 January 2016.

[bib27] Ding D., Gebel K. (2012). Built environment, physical activity, and obesity: what have we learned from reviewing the literature?. Health Place..

[bib28] Ding D., Sallis J.F., Kerr J., Lee S., Rosenberg D.E. (2011). Neighborhood environment and physical activity among youth a review. Am. J. Prev. Med..

[bib29] Dunton G.F., Kaplan J., Wolch J., Jerrett M., Reynolds K.D. (2009). Physical environmental correlates of childhood obesity: a systematic review. Obes. Rev..

[bib30] ESRI, 2014. ArcGIS 10.3 for Desktop.

[bib31] Evenson K.R., Wen F. (2013). Using geographic information systems to compare municipal, county, and commercial parks data. Prev. Chronic Dis..

[bib32] Feng J., Glass T.A., Curriero F.C., Stewart W.F., Schwartz B.S. (2010). The built environment and obesity: a systematic review of the epidemiologic evidence. Health Place..

[bib33] Fleischhacker S.E., Evenson K.R., Sharkey J., Pitts S.B., Rodriguez D.A. (2013). Validity of secondary retail food outlet data: a systematic review. Am. J. Prev. Med..

[bib34] Fleiss J.L. (1981). Statistical Methods for Rates and Proportions.

[bib35] Gasevic D., Vukmirovich I., Yusuf S., Teo K., Chow C., Dagenais G., Lear S.A. (2011). A direct assessment of "obesogenic" built environments: challenges and recommendations. J. Environ. Public Health.

[bib36] Giraudeau B. (1996). Negative values of the intraclass correlation coefficient are not theoretically possible. J. Clin. Epidemiol..

[bib37] Glanz K., Sallis J.F., Saelens B.E., Frank L.D. (2007). Nutrition Environment Measures Survey in stores (NEMS-S): development and evaluation. Am. J. Prev. Med..

[bib38] Goodwin D.M., Cummins S., Sautkina E., Ogilvie D., Petticrew M., Jones A., Wheeler K., White M. (2013). The role and status of evidence and innovation in the healthy towns programme in England: a qualitative stakeholder interview study. J. Epidemiol. Community Health.

[bib39] Gravlee C.C., Zenk S.N., Woods S., Rowe Z., Schulz A.J. (2006). Handheld computers for direct observation of the social and physical environment. Field Methods.

[bib40] Griew P., Hillsdon M., Foster C., Coombes E., Jones A., Wilkinson P. (2013). Developing and testing a street audit tool using Google street View to measure environmental supportiveness for physical activity. Int J. Behav. Nutr. Phys. Act..

[bib41] Hara, K., Le, V., Froehlich, J., 2013. Combining crowdsourcing and google street view to identify street-level accessibility problems. In: Proceedings of the SIGCHI Conference on Human Factors in Computing Systems. ACM Conference paper, Paris, France, pp. 631–640.

[bib42] Hara, K., Sun, J., Moore, R., Jacobs, D., Froehlich, J., 2014. Tohme: Detecting Curb Ramps in Google Street View Using Crowdsourcing, Computer Vision, and Machine Learning, SIGCHI Conference paper, pp. 189–204.

[bib43] Hawkesworth S., Silverwood R., Pliakas T., Nanchahal K., Jefferis B., Sartini C., Amuzu A., Armstrong B., Casas J.-P., Morris R., Whincup P., Lock K. (2015). How the local built environment affects physical activity behaviour in older adults in the UK: a cross-sectional analysis linked to two national cohorts. Lancet.

[bib44] King K.E. (2015). A comparison of two methods for measuring land use in public health research: systematic social observation versus remote sensing–based coded aerial photography. SAGE Open.

[bib45] Krenn P.J., Titze S., Oja P., Jones A., Ogilvie D. (2011). Use of global positioning systems to study physical activity and the environment: a systematic review. Am. J. Prev. Med..

[bib46] Lake A.A., Burgoine T., Stamp E., Grieve R. (2012). The foodscape: classification and field validation of secondary data sources across urban/rural and socio-economic classifications in England. Int J. Behav. Nutr. Phys. Act..

[bib47] Landis J.R., Koch G.G. (1977). The measurement of observer agreement for categorical data. Biometrics.

[bib48] Lawlor D.A., Bedford C., Taylor M., Ebrahim S. (2003). Geographical variation in cardiovascular disease, risk factors, and their control in older women: british women's Heart and Health Study. J. Epidemiol. Community Health.

[bib49] Lee R.E., Booth K.M., Reese-Smith J.Y., Regan G., Howard H.H. (2005). The physical activity resource assessment (para) instrument: evaluating features, amenities and incivilities of physical activity resources in urban neighborhoods. Int J. Behav. Nutr. Phys. Act..

[bib50] Leslie E., Coffee N., Frank L., Owen N., Bauman A., Hugo G. (2007). Walkability of local communities: using geographic information systems to objectively assess relevant environmental attributes. Health Place..

[bib51] Liese A.D., Barnes T.L., Lamichhane A.P., Hibbert J.D., Colabianchi N., Lawson A.B. (2013). Characterizing the food retail environment: impact of count, type, and geospatial error in 2 secondary data sources. J. Nutr. Educ. Behav..

[bib52] Local Government Association, 2013. A Guide to INSPIRE compliance in Local Government. Available at: 〈http://www.local.gov.uk/c/document_library/get_file?Uuid=ba19b779-eb8d-404c-af07-25ecd921aed9〉 (accessed 27.01.16). Archived at: 〈http://www.webcitation.org/6eqpOu6Gn〉 on 27 January 2016.

[bib53] Lucan S.C. (2015). Concerning limitations of food-environment research: a narrative review and commentary framed around obesity and diet-related diseases in youth. J. Acad. Nutr. Diet..

[bib54] Lucan S.C., Maroko A.R., Bumol J., Torrens L., Varona M., Berke E.M. (2013). Business list vs ground observation for measuring a food environment: saving time or waste of time (or worse)?. J. Acad. Nutr. Diet..

[bib55] Macintyre S. (2007). Deprivation amplification revisited; or, is it always true that poorer places have poorer access to resources for healthy diets and physical activity?. Int J. Behav. Nutr. Phys. Act..

[bib56] Macintyre S., Ellaway A., Cummins S. (2002). Place effects on health: how can we conceptualise, operationalise and measure them?. Soc. Sci. Med..

[bib57] Milton S., Pliakas T., Hawkesworth S., Nanchahal K., Grundy C., Amuzu A., Casas J.-P., Lock K. (2015). A qualitative geographical information systems approach to explore how older people over 70 years interact with and define their neighbourhood environment. Health Place..

[bib58] Office for National Statistics, 2015. Neighbourhood Statistics. Available at: 〈http://www.neighbourhood.statistics.gov.uk/dissemination/LeadHome.do〉 (accessed 27.01.16). Archived at: 〈http://www.webcitation.org/6eqqor1dR〉 on 27 January 2016.

[bib59] Paquet C., Daniel M., Kestens Y., Leger K., Gauvin L. (2008). Field validation of listings of food stores and commercial physical activity establishments from secondary data. Int J. Behav. Nutr. Phys. Act..

[bib60] Pikora T.J., Bull F.C., Jamrozik K., Knuiman M., Giles-Corti B., Donovan R.J. (2002). Developing a reliable audit instrument to measure the physical environment for physical activity. Am. J. Prev. Med..

[bib61] Pliakas T., Wilkinson P., Tonne C. (2014). Contribution of the physical environment to socioeconomic gradients in walking in the Whitehall II study. Health Place..

[bib62] Saelens B.E., Glanz K., Sallis J.F., Frank L.D. (2007). Nutrition Environment Measures Study in restaurants (NEMS-R): development and evaluation. Am. J. Prev. Med..

[bib63] Saelens B.E., Handy S.L. (2008). Built environment correlates of walking: a review. Med. Sci. Sports Exerc.

[bib64] Sallis J.F., Cervero R.B., Ascher W., Henderson K.A., Kraft M.K., Kerr J. (2006). An ecological approach to creating active living communities. Annu Rev. Public Health.

[bib65] Sampson R.J., Raudenbush S.W. (1999). Systematic Social Observation of Public Spaces: a New Look at Disorder in Urban Neighborhoods. Am. J. Sociol..

[bib66] Schaefer-McDaniel N., Caughy M.O., O'Campo P., Gearey W. (2010). Examining methodological details of neighbourhood observations and the relationship to health: a literature review. Soc. Sci. Med.

[bib67] Shareck M., Dassa C., Frohlich K.L. (2012). Improving the measurement of neighbourhood characteristics through systematic observation: inequalities in smoking as a case study. Health Place..

[bib68] Shrout P.E., Fleiss J.L. (1979). Intraclass correlations: uses in assessing rater reliability. Psychol. Bull..

[bib69] StataCorp LP, 2015. Stata/IC 14.1.

[bib70] Thornton L.E., Pearce J.R., Kavanagh A.M. (2011). Using Geographic Information Systems (GIS) to assess the role of the built environment in influencing obesity: a glossary. Int J. Behav. Nutr. Phys. Act..

[bib71] Van Cauwenberg J., De Bourdeaudhuij I., De Meester F., Van Dyck D., Salmon J., Clarys P., Deforche B. (2011). Relationship between the physical environment and physical activity in older adults: a systematic review. Health Place..

[bib72] Walker M., Whincup P.H., Shaper A.G. (2004). The British regional heart study 1975–2004. Int J. Epidemiol..

[bib73] Wilson J.S., Kelly C.M., Schootman M., Baker E.A., Banerjee A., Clennin M., Miller D.K. (2012). Assessing the built environment using omnidirectional imagery. Am. J. Prev. Med.

[bib74] World Health Organization, 2010. Urban planning, environment and health. Available at: 〈http://www.euro.who.int/__data/assets/pdf_file/0004/114448/E93987.pdf〉 (accessed 27.01.16). Archived at: 〈http://www.webcitation.org/6eqpesahM〉 on 27 January 2016.

[bib75] Wu Y.T., Nash P., Barnes L.E., Minett T., Matthews F.E., Jones A., Brayne C. (2014). Assessing environmental features related to mental health: a reliability study of visual streetscape images. BMC Public Health.

[bib76] Zenk S.N., Schulz A.J., Mentz G., House J.S., Gravlee C.C., Miranda P.Y., Miller P., Kannan S. (2007). Inter-rater and test-retest reliability: methods and results for the neighborhood observational checklist. Health Place..

