# Supplementary material for: Optimising measurement of health-related characteristics of the built environment: Comparing data collected by foot-based street audits, virtual street audits and routine secondary data sources
Source: Health Place. 2017 Jan;43:75–84. doi: 10.1016/j.healthplace.2016.10.001 (PMC5292100; doi:10.1016/j.healthplace.2016.10.001)
Supplement: Supplementary file 1 — Supplementary material [file mmc1.docx]

# Appendix S1. ‘Older People’s Environments and CVD Risk’ (OPECR) tool

| ***Area (SOA):*** | ***Date & Time:*** | ***Observer:*** |
| --- | --- | --- |
| ***Segment:*** |  | *Small segment* |
| ***Start point:*** |  | *Medium segment* |
| ***End point:*** |  | *Large segment* |

| ***Traffic volume (in 5 minutes)*** |  |  |
| --- | --- | --- |
| Number of motorised vehicles **(not buses)** passing |  | ***Total:*** |
| Buses |  |  |

| ***Built environment – Circle*** | | | | | | |
| --- | --- | --- | --- | --- | --- | --- |
| **Pavements Quality** | Poor | Fair | Good | Excellent | | N/A |
| **Lowered curbs** | None | Few / Poor | Most | All | | N/A |
| **Barriers on pavement** | None | Occasional | Often |  | | N/A |
| **Pavement width – Left** | None/too narrow for 1 person | 1 person / variable | 2 people | >2 people | |  |
| **Pavement width – Right** | None/too narrow for 1 person | 1 person / variable | 2 people | >2 people | |  |
| **Pedestrian traffic** | No people | Few/occasional | Many/often | Crowded | |  |
| **Road use** | One way only | 2 way without lane markings | 2 way with lane markings | 3+ lanes | | N/A |
| **Road connectivity** | Through road for traffic | Cul de Sac | Cul de sac with pedestrian throughway | Pedestrianalised | |  |
| **Traffic calming measures** | Absent | At 1 or 2 points | Regularly along segment | N/A | **SPEED LIMIT:** | |
| **Parking spaces available** | None | Mostly restricted | Several sections of unrestricted | Unlimited | | N/A |
| **Parked cars** | None | Only few | Bays approx half filled | Parking filled +/or obstruction of crossings | | N/A |
| **Lamp posts**  **(is it well lit?)** | None | Sporadically placed/damaged | Spaced at regular intervals one side | Spaced at regular intervals both side | |  |
| **Slope** | Flat | Slight hill | Steep hill |  | |  |

| ***Crossings - tally*** |  | ***Amenities - tally*** |  |
| --- | --- | --- | --- |
| Traffic lights with pedestrian indicators |  | Benches |  |
| Zebra/pelican crossings |  | Public toilets |  |
| Traffic lights no indicators |  | Post boxes |  |
| Lowered curb/ middle road island |  | Phone booths |  |
| Pedestrian underpass/ overpass |  | Small public green/paved area |  |
|  |  | Access to Large Park (mark on map) |  |
| ***Transport - tally*** |  | Walking Trails |  |
| Bus stop with shelter |  | Alley / connective Footpath |  |
| Bus stop without shelter |  | Public bins |  |
| Disabled parking bays |  | No. of locations of recycling bins |  |
| Taxi rank |  | No. of locations of commercial bins |  |

| ***Aesthetics*** | **None** | **Few** | **Many** |  |
| --- | --- | --- | --- | --- |
| Neighbourhood watch/residential signs |  |  |  |  |
| Security Measures e.g. Barred windows |  |  |  |  |
|  | **None** | **Little** | **Moderate** | **Extensive** |
| Greenery factors |  |  |  |  |
| Graffiti |  |  |  |  |
| Litter, dog foul, broken glass etc |  |  |  |  |

| *Date & Time:* | *Segment:* | *Observer:* |
| --- | --- | --- |

| ***Shops & Services density – tally*** |  |  |  |
| --- | --- | --- | --- |
| Independent Convenience/General stores |  | Other non food shops/services |  |
| Small Supermarkets |  | Pharmacy |  |
| Large Supermarkets with parking |  | GP |  |
| Off-Licences |  | Dentist - NHS |  |
| Fast Food outlets (eat in or take away) |  | Dentist - Private |  |
| Restaurants (other sit down) |  | Hospital |  |
| Other Food Shops |  | Other healthcare e.g. Opticians |  |
| Cafes, coffee shops, sandwich shops –  NO outdoor seating area |  | Residential Homes for older people |  |
| Cafes, coffee shops, sandwich shops –  UNCOVERED outdoor seated area |  | Religious venue, Community Centre, Advice bureau, School/college, Library |  |
| cafes, coffe shops, sandwich shops –  SHELTERED outdoor seating area |  | Leisure centres, Exercise venues |  |
| Pubs/Bars - NO outdoor seating |  | Public funded swimming pools |  |
| Pubs/Bars - UNCOVERED outdoor seating |  | Laundrettes & Hairdressers etc |  |
| Pubs/Bars - SHELTERED outdoor seating |  | Banks & Post offices |  |
| Hotel, B & B (accommodation) |  | Recreational venues e.g betting shops, bingo, museums, art galleries |  |
|  |  | Shopping centre/mall (access to) |  |

| ***Shops selling: - tally*** | All Poor quality | Medium | All Good  Quality |  | ***Prices:* - *write in*** | Supermarket | Local stores |
| --- | --- | --- | --- | --- | --- | --- | --- |
| Fruit & veg < 5 variety |  |  |  |  | 20 pack cigarettes cheapest |  |  |
| Fruit & veg ≥ 5 variety |  |  |  |  | 20 pack cigarettes e.g. Marlboro light |  |  |
|  |  |  |  |  | 1 loaf white bread |  |  |
| Alcohol |  |  |  |  | 1pt full fat milk |  |  |
| Cigarettes |  |  |  |  | 1kg carrots |  |  |
|  |  |  |  |  | 1kg apples (or state) |  |  |
| **Tick if 'DESTINATION'**  **present in segment** | |  |  |  | Cheapest 1L Vodka |  |  |
|  |  |  |  |  | Cheapest Can Beer |  |  |

| ***Landuse*** | **Predominant buildings**  ***(tick 1)*** | **Next major contributor**  ***(tick 1)*** | **Tick if present in segment** |
| --- | --- | --- | --- |
| Purpose built block of flats <10flrs |  |  |  |
| High rise block of flats >10floors |  |  |  |
| Offices |  |  |  |
| Shops & services, schools |  |  |  |
| Offices/shops with flats above |  |  |  |
| Industrial/Other Commercial Buildings/Car Parks |  |  |  |
| Terraced houses |  |  |  |
| Detached or Semi-detached houses |  |  |  |
| Green areas (parks, fields, hedges) |  |  |  |
| Derelict or vacant building or plot |  |  |  |

| ***Adverts - tally*** | **Shop Windows** | **Billboards** | **Other** |
| --- | --- | --- | --- |
| Alcoholic drinks |  |  |  |
| Sugary drinks |  |  |  |
| Unhealthy snacks/ junk food |  |  |  |
| Smoking cessation |  |  |  |
| Commercial promotion of healthy foods, drinks |  |  |  |
| Non-commercial promotion e.g. healthy diets, weight loss |  |  |  |
| Commercial promoting physical activity e.g. gyms, health centres |  |  |  |
| Non- commercial promoting physical activity e.g. free swim |  |  |  |

| **General notes:** |
| --- |
