# Supplementary material for: Optimising measurement of health-related characteristics of the built environment: Comparing data collected by foot-based street audits, virtual street audits and routine secondary data sources
Source: Health Place. 2017 Jan;43:75–84. doi: 10.1016/j.healthplace.2016.10.001 (PMC5292100; doi:10.1016/j.healthplace.2016.10.001)
Supplement: Supplementary file 2 — Supplementary material [file mmc2.docx]

**Appendix S2.** Pearson correlation between foot based street and Google Street View audits.

| **Variable** | **Pearson r** |
| --- | --- |
| Number of traffic lights with pedestrian indicators | 0.76 |
| Number of Zebra or Pelican crossings | 0.55 |
| Number of traffic lights without pedestrian indicators | 0.43 |
| Number of lowered curbs, or traffic islands | 0.66 |
| Number of under passes, over passes or bridges | 0.73 |
| Road crossings (traffic lights, zebra crossings, lowered curbs and under passes | 0.76 |
| Number of bus stops with shelter | 0.84 |
| Number of bus stops without shelter | 0.76 |
| Bus stops | 0.80 |
| Number of disabled parking bays | 0.30 |
| Number of taxi ranks | 0.00 |
| Number of benches | 0.66 |
| Number of public toilets | 0.51 |
| Number of post boxes | 0.75 |
| Number of phone boxes | 0.72 |
| Number of public bins | 0.56 |
| Number of recycling locations | 0.04 |
| Number of commercial bins | 0.21 |
| Amenities (Benches, toilets, post and phone boxes, bins and recycling locations) | 0.67 |
| Number of small green/paved areas | 0.23 |
| Number of access points to large parks | 0.38 |
| Access to green space (Small green/paved areas and access points to large parks) | 0.35 |
| Number of walking trails | 0.27 |
| Number of alleys / connecting footpaths | 0.34 |
| Trails and footpaths (Walking trails and alleys) | 0.37 |
| Number of independent convenience stores | 0.49 |
| Number of small supermarkets | 0.61 |
| Number of large supermarkets with parking | 0.53 |
| Number of off-licence | 0.20 |
| Number of fast food outlet | 0.65 |
| Number of restaurants | 0.49 |
| Number of other food shops | 0.73 |
| Number of cafes without smoking area | 0.55 |
| Number of cafes with outside smoking area with no shelter available | 0.19 |
| Number of cafes with outside smoking area with shelter available ^a^ | - |
| Number of pubs without smoking area | 0.57 |
| Number of pubs with outside smoking area with no shelter available | 0.46 |
| Number of pubs with outside smoking area with shelter available | 0.16 |
| Number of hotels | 0.35 |
| Number of non-food shops | 0.85 |
| Number of pharmacy | 0.65 |
| Number of GP | 0.35 |
| Number of NHS dentists | 0.20 |
| Number of private dentists | 0.16 |
| Number of hospitals | 0.34 |
| Number of other healthcare | 0.10 |
| Number of residential homes | 0.58 |
| Number of religious centres | 0.46 |
| Number of leisure centres | 0.31 |
| Number of public swimming pools ^a^ | - |
| Number of laundrettes and hairdressers | 0.76 |
| Number of banks and post offices | 0.90 |
| Number of recreational venues | 0.38 |
| Number of shopping centres ^a^ | - |
| Shops and services (Convenience stores, supermarkets, off-license, fast food out | 0.90 |
| Number of alcoholic drinks advert (in shop windows) | 0.33 |
| Number of sugary drinks advert (in shop windows) | 0.00 |
| Number of snack/junk food adverts (in shop windows) | 0.13 |
| Number of smoking cessation adverts (in shop windows) ^a^ | - |
| Number of commercial healthy food adverts (in shop windows) | 0.00 |
| Number of non-commercial food adverts (in shop windows) ^a^ | - |
| Number of commercial physical activity adverts (in shop windows) ^a^ | - |
| Number of non-commercial physical activity adverts (in shop windows) ^a^ | - |
| Adverts in shop windows (Alcoholic, sugary, snack/junk food, smoking cessation, | 0.30 |
| Number of alcoholic drinks advert (on bill boards) ^a^ | - |
| Number of sugary drinks advert (on bill boards) ^a^ | - |
| Number of snack/junk food adverts (on bill boards) ^a^ | - |
| Number of smoking cessation adverts (on bill boards) ^a^ | - |
| Number of commercial healthy food adverts (on bill boards) ^a^ | - |
| Number of non-commercial food adverts (on bill boards) ^a^ | - |
| Number of commercial physical activity adverts (on bill boards) ^a^ | - |
| Number of non-commercial physical activity adverts (on bill boards) ^a^ | - |
| Adverts on bill boards (Alcoholic, sugary, snack/junk food, smoking cessation, etc) ^a^ | - |
| Number of alcoholic drinks advert (other) | 0.00 |
| Number of sugary drinks advert (other) ^a^ | - |
| Number of snack/junk food adverts (other) | 0.08 |
| Number of smoking cessation adverts (other) ^a^ | - |
| Number of commercial healthy food adverts (other) | 0.00 |
| Number of non-commercial food adverts (other) ^a^ | - |
| Number of commercial physical activity adverts (other) ^a^ | - |
| Number of non-commercial physical activity adverts (other) ^a^ | - |
| Adverts (other - Alcoholic, sugary, snack/junk food, smoking cessation, etc) | 0.02 |

^a^ No variation in Google Street View ratings.

GP: General Practitioner.
