# Supplementary material for: Optimising measurement of health-related characteristics of the built environment: Comparing data collected by foot-based street audits, virtual street audits and routine secondary data sources
Source: Health Place. 2017 Jan;43:75–84. doi: 10.1016/j.healthplace.2016.10.001 (PMC5292100; doi:10.1016/j.healthplace.2016.10.001)
Supplement: Supplementary file 3 — Supplementary material [file mmc3.docx]

Appendix S3. Latent Class Analyses

# Materials and Methods

## Measures

In both the foot-based audit and Street View latent class analyses (LCAs) the following 10 built environment variables were used: ‘quality of pavement’, ‘lowered curbs’, ‘barriers on pavement’, ‘pavement width’, ‘pedestrian traffic’, ‘road use’, ‘road connectivity’, ‘traffic calming measures’, ‘lamp posts’ and ‘road crossings’. The first 9 of these variables were originally collected as 4- or 5-level categorical variables and were collapsed to 3-level categorical variables to avoid small numbers (or to binary variables if small numbers were still unavoidable). ‘Road crossings’ was originally collected as a count of the number of crossings. As the majority of segments had no crossings this was analysed as ‘none’ vs. ‘one or more’. Segments with data on at least 7 of the 10 built environment variables were included in the LCAs.

## Statistical analysis

LCA is a multivariate regression approach that describes the relationship between an unobserved categorical latent variable, each level of which is a ‘latent class’, and a set of observed dependent variables (‘latent class indicators’), in this case the ten built environment variables. The resultant latent classes identify subgroups that have similar patterns of the latent class indicators (natural clusterings of features of the built environment in this instance). The objective is usually to identify the latent class indicators that best distinguish between classes and to categorise the units of analysis into their most likely classes given their observed responses (1).

LCA was conducted on the foot-based audit and Street View data separately. The expectation-maximization (EM) algorithm (2) was used to allow the inclusion of segments with incomplete built environment data. The EM algorithm relies on the assumption that data are missing at random (3). To ensure a true (rather than local) maximum likelihood solution had been reached we used 100, 1,000 or 10,000 random start values (as necessary) for the estimation of each model.

We used several different tools to decide how many classes were appropriate as no single approach is agreed upon as optimal (1). The standard likelihood ratio test (LRT) is invalid in the LCA setting, so we used the Lo-Mendell-Rubin adjusted LRT (4). We also used three information criteria: Akaike’s Information Criterion (AIC) (5), Schwarz’s Bayesian Information Criterion (BIC) (6), and sample size-adjusted BIC (aBIC) (7). Models that best combine goodness-of-fit and parsimony are indicated by lower values of the information criteria. In addition, the relative class sizes (calculated based on most likely class membership), meaningful interpretation of the latent classes and entropy were considered. Entropy is a summary statistic, based on the posterior class membership probabilities, which evaluates the clarity of the separation of the latent classes, with values ranging from 0 (unclear classification) to 1 (clear classification) (8). The LCAs were conducted using Mplus 6 (9).

# Results

## Foot-based audit

After the exclusion of cul-de-sacs, 16,520 segments remained and were eligible for inclusion in the foot-based audit LCA. Of these, 16,010 (96.9%) segments with data on at least 7 of the 10 built environment variables (14,909 with complete data on all 10 variables) were included in the LCA. The distributions of the foot-based audit built environment variables are given in Table S3a. Model fit statistics for the foot-based audit LCA are given in Table S3b. Although the Lo-Mendell-Rubin LRT suggested continuing to add more classes, the information criteria showed only moderate decreases beyond 3 classes. The 3-class model also provided more easily interpretable classes and with higher entropy that models with more than 3 classes. On the balance of evidence the 3-class model was chosen as most appropriate. The class-specific latent class indicator probabilities for the foot-based audit LCA are given in Table S3c. Class 1 (9.9%) was more likely to have no pavements, have no pedestrian traffic, be a cul de sac with pedestrian throughway/pedestrianised, have no/sporadically/damaged lamp posts, and have no street crossings; class 2 (57.0%) was more likely to have poor/fair quality pavement, have no/few/poor lowered curbs, have medium width pavement, have no pedestrian traffic, have no traffic calming measures, have regular lamp posts on one side, and have no road crossings; and class 3 (33.1%) was more likely to have good/excellent quality pavement, have most/all lowered curbs, have no barriers on the pavement, have wide pavement, have more pedestrian traffic, be a through road, have regular traffic calming measures, have regular lamp posts on both sides, and have one or more road crossings.

## Street View data

After the exclusion of cul-de-sacs, 2051 segments remained and were eligible for inclusion in the Street View LCA. Of these, 1352 (65.9%) segments with data on at least 7 of the 10 built environment variables (1292 with complete data on all 10 variables) were included in the LCA. The distributions of the Street View built environment variables are given in Table S3d.

Model fit statistics for the Street View LCA are given in Table S3e. The Lo-Mendell-Rubin LRT suggested that the 3-class model provided a better fit than the 2-class model but that there was no need to add any further classes. The information criteria showed only moderate decreases beyond 3 classes. The 3-class model also provided more easily interpretable classes and with higher entropy that models with more than 3 classes. The 3-class model was therefore again chosen as most appropriate.

The class-specific latent class indicator probabilities for the Street View LCA are given in Table S3f. Class 1 (41.0%) was more likely to have good/excellent quality pavement, have most/all lowered curbs, have no barriers on pavement, have wide pavement, have more pedestrian traffic, be a through road, have regular traffic calming measures, have regular lamp posts on both sides, and have one or more road crossings; class 2 (4.5%) was more likely to have no pavements, have no pedestrian traffic, be a cul de sac with pedestrian throughway/pedestrianised, have no/sporadically/damaged lamp posts, and have no street crossings; and class 3 (54.5%) was more likely to have no/few/poor lowered curbs, have medium width pavement, have no pedestrian traffic, be a cul de sac with pedestrian throughway/pedestrianised, have regular lamp posts on one side, and have no road crossings.

The characteristics of the latent classes in the foot-based audit and Street View LCAs were therefore very similar, with class 1 in the foot-based audit LCA equivalent to class 2 in the Street View LCA; class 2 in the foot-based audit LCA equivalent to class 3 in the Street View LCA; and class 3 in the foot-based audit LCA equivalent to class 1 in the Street View LCA.

# References

1. Nylund KL, Asparouhov T, Muthen BO. Deciding on the Number of Classes in Latent Class Analysis and Growth Mixture Modeling: A Monte Carlo Simulation Study. Struct Equ Modeling. 2007; 14(4): 535-569.

2. Dempster AP, Laird NM, Rubin DB. Maximum likelihood from incomplete data via the EM algorithm. J R Stat Soc Series B Stat Methodol. 1977; 39(1): 1-38.

3. Little RJA, Rubin DB. Statistical Analysis With Missing Data. New York: Wiley; 2002.

4. Lo Y, Mendell NR, Rubin DB. Testing the number of components in a normal mixture. Biometrika. 2001; 88(3): 767-778.

5. Akaike H. Factor analysis and AIC. Psychometrika. 1987; 52(3): 317-332.

6. Schwartz G. Estimating the dimension of a model. Ann Stat. 1978; 6(2): 461-464.

7. Sclove L. Application of model-selection criteria to some problems in multivariate analysis. Psychometrika. 1987; 52(3): 333-343.

8. Muthén LK, Muthén BO. Mplus User’s Guide. Seventh Edition. Los Angeles, CA: Muthén & Muthén; 1998-2012.

9. Muthén & Muthén. Mplus statistical software, release 6. Los Angeles, CA: Muthén & Muthén; 2010.

# Tables

Table S3a. Foot-based audit built environment data (n = 16,010).

|  | n | % |
| --- | --- | --- |
| Quality of pavement |  |  |
| Poor/fair | 6928 | 43.5 |
| Good/excellent | 7448 | 46.7 |
| N/A | 1564 | 9.8 |
| Total | 15,940 |  |
|  |  |  |
| Lowered curbs |  |  |
| None/few/poor | 6060 | 38.7 |
| Most/all | 7985 | 51.0 |
| N/A | 1599 | 10.2 |
| Total | 15,644 |  |
|  |  |  |
| Barriers on pavement |  |  |
| None | 10,377 | 65.5 |
| Occasional/often | 3908 | 24.7 |
| N/A | 1555 | 9.8 |
| Total | 15,840 |  |
|  |  |  |
| Pavement width |  |  |
| None/narrow/1 person/variable | 3509 | 21.9 |
| 2 people | 10,159 | 63.5 |
| > 2 people | 2323 | 14.5 |
| Total | 15,991 |  |
|  |  |  |
| Pedestrian traffic |  |  |
| No people | 4213 | 26.5 |
| Few | 10,628 | 66.9 |
| Many/crowded | 1044 | 6.6 |
| Total | 15,885 |  |
|  |  |  |
| Road use |  |  |
| One-way | 506 | 3.2 |
| Two-way | 15,280 | 96.8 |
| Total | 15,786 |  |
|  |  |  |
| Road connectivity |  |  |
| Through road | 12,320 | 77.1 |
| Cul de sac with pedestrian throughway/pedestrianised | 3658 | 22.9 |
| Total | 15,978 |  |
|  |  |  |
| Traffic calming measures |  |  |
| Absent | 12,758 | 81.1 |
| At one or two points | 1813 | 11.5 |
| Regularly | 1166 | 7.4 |
| Total | 15,737 |  |
|  |  |  |
| Lamp posts |  |  |
| None/sporadically/damaged | 2333 | 14.6 |
| Regularly on one side | 3881 | 24.4 |
| Regularly on both sides | 9713 | 61.0 |
| Total | 15,927 |  |
|  |  |  |
| Road crossings |  |  |
| None | 13,596 | 84.9 |
| One or more | 2410 | 15.1 |
| Total | 16,006 |  |

Table S3b. Foot-based audit built environment latent class analysis model fit statistics. Full information maximum likelihood (n = 16,010).

|  | 1  class | 2  classes | 3  classes | 4  classes | 5  classes | 6  classes |
| --- | --- | --- | --- | --- | --- | --- |
| No. parameters | 17 | 35 | 53 | 71 | 89 | 107 |
| Loglikelihood | -112,430 | -98,760 | -96,598 | -96,069 | -95,705 | -95,479 |
|  |  |  |  |  |  |  |
| Information criterion |  |  |  |  |  |  |
| AIC | 224,894 | 197,589 | 193,303 | 192,280 | 191,589 | 191,172 |
| BIC | 225,025 | 197,858 | 193,708 | 192,825 | 192,272 | 191,994 |
| aBIC | 224,971 | 197,747 | 193,541 | 192,600 | 191,989 | 191,654 |
|  |  |  |  |  |  |  |
| Entropy | - | 0.997 | 0.727 | 0.643 | 0.642 | 0.641 |
|  |  |  |  |  |  |  |
| Minimum class proportion | - | 0.099 | 0.099 | 0.098 | 0.075 | 0.080 |
|  |  |  |  |  |  |  |
| Lo-Mendell-Rubin LRT p-value | - | <0.001 | <0.001 | <0.001 | <0.001 | <0.001 |

AIC, Akaike’s Information Criterion; BIC, Bayesian Information Criterion; aBIC, sample size adjusted Bayesian Information Criterion, LRT, likelihood ratio test.

Table S3c. Foot-based audit class-specific latent class indicator probabilities (3 classes).

|  | Observed  proportion | Class 1  (9.9%) | Class 2  (57.0%) | Class 3  (33.1%) |
| --- | --- | --- | --- | --- |
| Quality of pavement |  |  |  |  |
| Poor/fair | 0.435 | 0.008 | 0.570 | 0.345 |
| Good/excellent | 0.467 | 0.014 | 0.428 | 0.655 |
| N/A | 0.098 | 0.978 | 0.002 | 0.000 |
|  |  |  |  |  |
| Lowered curbs |  |  |  |  |
| None/few/poor | 0.387 | 0.012 | 0.584 | 0.194 |
| Most/all | 0.510 | 0.029 | 0.408 | 0.804 |
| N/A | 0.102 | 0.959 | 0.008 | 0.002 |
|  |  |  |  |  |
| Barriers on pavement |  |  |  |  |
| None | 0.655 | 0.012 | 0.706 | 0.758 |
| Occasional/often | 0.247 | 0.005 | 0.294 | 0.242 |
| N/A | 0.098 | 0.983 | 0.000 | 0.000 |
|  |  |  |  |  |
| Pavement width |  |  |  |  |
| None/narrow/1 person/variable | 0.219 | 0.987 | 0.164 | 0.092 |
| 2 people | 0.635 | 0.004 | 0.758 | 0.621 |
| > 2 people | 0.145 | 0.010 | 0.078 | 0.287 |
|  |  |  |  |  |
| Pedestrian traffic |  |  |  |  |
| No people | 0.265 | 0.557 | 0.328 | 0.086 |
| Few | 0.669 | 0.409 | 0.658 | 0.759 |
| Many/crowded | 0.066 | 0.034 | 0.014 | 0.155 |
|  |  |  |  |  |
| Road use |  |  |  |  |
| One-way | 0.038 | 0.038 | 0.020 | 0.049 |
| Two-way | 0.962 | 0.962 | 0.980 | 0.951 |
|  |  |  |  |  |
| Road connectivity |  |  |  |  |
| Through road | 0.771 | 0.632 | 0.674 | 0.960 |
| Cul de sac with pedestrian throughway/pedestrianised | 0.229 | 0.368 | 0.326 | 0.040 |
|  |  |  |  |  |
| Traffic calming measures |  |  |  |  |
| Absent | 0.811 | 0.856 | 0.897 | 0.666 |
| At one or two points | 0.115 | 0.091 | 0.075 | 0.184 |
| Regularly | 0.074 | 0.053 | 0.028 | 0.150 |
|  |  |  |  |  |
| Lamp posts |  |  |  |  |
| None/sporadically/damaged | 0.146 | 0.686 | 0.125 | 0.030 |
| Regularly on one side | 0.244 | 0.185 | 0.317 | 0.147 |
| Regularly on both sides | 0.610 | 0.130 | 0.558 | 0.824 |
|  |  |  |  |  |
| Road crossings |  |  |  |  |
| None | 0.849 | 0.970 | 0.976 | 0.620 |
| One or more | 0.151 | 0.030 | 0.024 | 0.380 |

Table S3d. Google Street View built environment data (n = 1352).

|  | n | % |
| --- | --- | --- |
| Quality of pavement |  |  |
| Poor/fair | 285 | 21.1 |
| Good/excellent | 998 | 73.8 |
| N/A | 69 | 5.1 |
| Total | 1352 |  |
|  |  |  |
| Lowered curbs |  |  |
| None/few/poor | 497 | 36.8 |
| Most/all | 798 | 59.0 |
| N/A | 57 | 4.2 |
| Total | 1352 |  |
|  |  |  |
| Barriers on pavement |  |  |
| None | 930 | 68.8 |
| Occasional/often | 361 | 26.7 |
| N/A | 61 | 4.5 |
| Total | 1352 |  |
|  |  |  |
| Pavement width |  |  |
| None/narrow/1 person/variable | 208 | 15.4 |
| 2 people | 930 | 68.8 |
| > 2 people | 214 | 15.8 |
| Total | 1351 |  |
|  |  |  |
| Pedestrian traffic |  |  |
| No people | 603 | 44.6 |
| Few | 643 | 47.6 |
| Many/crowded | 106 | 7.8 |
| Total | 1352 |  |
|  |  |  |
| Road use |  |  |
| One-way | 33 | 2.5 |
| Two-way | 1299 | 97.5 |
| Total | 1352 |  |
|  |  |  |
| Road connectivity |  |  |
| Through road | 1020 | 76.2 |
| Cul de sac with pedestrian throughway/pedestrianised | 318 | 23.8 |
| Total | 1338 |  |
|  |  |  |
| Traffic calming measures |  |  |
| Absent | 1150 | 85.6 |
| At one or two points | 136 | 10.1 |
| Regularly | 58 | 4.3 |
| Total | 1344 |  |
|  |  |  |
| Lamp posts |  |  |
| None/sporadically/damaged | 87 | 6.6 |
| Regularly on one side | 541 | 40.8 |
| Regularly on both sides | 697 | 52.6 |
| Total | 1325 |  |
|  |  |  |
| Road crossings |  |  |
| None | 1128 | 83.4 |
| One or more | 224 | 16.6 |
| Total | 1352 |  |

Table S3e. Google Street View built environment latent class analysis model fit statistics. Full information maximum likelihood (n = 1352).

|  | 1  class | 2  classes | 3  classes | 4  classes | 5  classes | 6  classes |
| --- | --- | --- | --- | --- | --- | --- |
| No. parameters | 17 | 35 | 53 | 71 | 89 | 107 |
| Loglikelihood | -8768 | -8255 | -8077 | -8013 | -7981 | -7952 |
|  |  |  |  |  |  |  |
| Information criterion |  |  |  |  |  |  |
| AIC | 17,571 | 16,579 | 16,259 | 16,169 | 16,140 | 16,118 |
| BIC | 17,659 | 16,762 | 16,536 | 16,539 | 16,604 | 16,676 |
| aBIC | 17,605 | 16,650 | 16,367 | 16,313 | 16,321 | 16,336 |
|  |  |  |  |  |  |  |
| Entropy | - | 0.996 | 0.694 | 0.633 | 0.615 | 0.611 |
|  |  |  |  |  |  |  |
| Minimum class proportion | - | 0.044 | 0.045 | 0.043 | 0.043 | 0.042 |
|  |  |  |  |  |  |  |
| Lo-Mendell-Rubin LRT p-value | - | <0.001 | 0.02 | 1.00 | 0.27 | 1.00 |

AIC, Akaike’s Information Criterion; BIC, Bayesian Information Criterion; aBIC, sample size adjusted Bayesian Information Criterion, LRT, likelihood ratio test.

Table S3f. Google Street View built environment class-specific latent class indicator probabilities (3 classes).

|  | Observed  proportion | Class 1  (41.0%) | Class 2  (4.5%) | Class 3  (54.5%) |
| --- | --- | --- | --- | --- |
| Quality of pavement |  |  |  |  |
| Poor/fair | 0.211 | 0.168 | 0.000 | 0.262 |
| Good/excellent | 0.738 | 0.827 | 0.049 | 0.726 |
| N/A | 0.051 | 0.005 | 0.951 | 0.011 |
|  |  |  |  |  |
| Lowered curbs |  |  |  |  |
| None/few/poor | 0.368 | 0.270 | 0.043 | 0.472 |
| Most/all | 0.590 | 0.728 | 0.105 | 0.522 |
| N/A | 0.042 | 0.002 | 0.851 | 0.006 |
|  |  |  |  |  |
| Barriers on pavement |  |  |  |  |
| None | 0.688 | 0.743 | 0.035 | 0.699 |
| Occasional/often | 0.267 | 0.257 | 0.000 | 0.298 |
| N/A | 0.045 | 0.000 | 0.965 | 0.003 |
|  |  |  |  |  |
| Pavement width |  |  |  |  |
| None/narrow/1 person/variable | 0.154 | 0.090 | 0.918 | 0.140 |
| 2 people | 0.688 | 0.633 | 0.033 | 0.786 |
| > 2 people | 0.158 | 0.277 | 0.049 | 0.074 |
|  |  |  |  |  |
| Pedestrian traffic |  |  |  |  |
| No people | 0.446 | 0.186 | 0.688 | 0.631 |
| Few | 0.476 | 0.648 | 0.248 | 0.359 |
| Many/crowded | 0.078 | 0.166 | 0.065 | 0.010 |
|  |  |  |  |  |
| Road use |  |  |  |  |
| One-way | 0.025 | 0.042 | 0.034 | 0.010 |
| Two-way | 0.975 | 0.958 | 0.966 | 0.990 |
|  |  |  |  |  |
| Road connectivity |  |  |  |  |
| Through road | 0.762 | 0.962 | 0.548 | 0.622 |
| Cul de sac with pedestrian throughway/pedestrianised | 0.238 | 0.038 | 0.452 | 0.378 |
|  |  |  |  |  |
| Traffic calming measures |  |  |  |  |
| Absent | 0.856 | 0.824 | 0.767 | 0.888 |
| At one or two points | 0.101 | 0.097 | 0.183 | 0.098 |
| Regularly | 0.043 | 0.079 | 0.050 | 0.015 |
|  |  |  |  |  |
| Lamp posts |  |  |  |  |
| None/sporadically/damaged | 0.066 | 0.021 | 0.450 | 0.069 |
| Regularly on one side | 0.408 | 0.311 | 0.294 | 0.494 |
| Regularly on both sides | 0.526 | 0.668 | 0.257 | 0.437 |
|  |  |  |  |  |
| Road crossings |  |  |  |  |
| None | 0.834 | 0.622 | 0.951 | 0.992 |
| One or more | 0.166 | 0.378 | 0.049 | 0.008 |
