# Supplementary material for: Optimising measurement of health-related characteristics of the built environment: Comparing data collected by foot-based street audits, virtual street audits and routine secondary data sources
Source: Health Place. 2017 Jan;43:75–84. doi: 10.1016/j.healthplace.2016.10.001 (PMC5292100; doi:10.1016/j.healthplace.2016.10.001)
Supplement: Supplementary file 4 — Supplementary material [file mmc4.docx]

**Appendix S4.** Summary statistics for built environment, aesthetics, and land use variables and Google Street View quality issues at segment level (n=1396, unless otherwise stated)^a^.

|  | **Foot-based audits** | | **Google Street View audits** | |
| --- | --- | --- | --- | --- |
| **Variable** | **N** | **%** | **N** | **%** |
| ***Built environment*** |  |  |  |  |
| Walkability index |  |  |  |  |
| Poor walking environment | 103 | 8.10 | 56 | 4.41 |
| Medium walking environment | 735 | 57.83 | 690 | 54.29 |
| Good walking environment | 433 | 34.07 | 525 | 41.31 |
| Quality of pavement (n=1,377_FBA_) |  |  |  |  |
| Poor | 50 | 3.63 | 13 | 0.93 |
| Fair | 489 | 35.51 | 281 | 20.13 |
| Good | 665 | 48.29 | 784 | 56.16 |
| Excellent | 102 | 7.41 | 238 | 17.05 |
| N/A | 71 | 5.16 | 80 | 5.73 |
| What proportion of curbs lowered in the appropriate places (n=1,333 _FBA_) |  |  |  |  |
| None | 213 | 15.98 | 336 | 24.07 |
| Few/poor | 163 | 12.23 | 173 | 12.39 |
| Most | 366 | 27.46 | 280 | 20.06 |
| All | 519 | 38.93 | 544 | 38.97 |
| N/A | 72 | 5.40 | 63 | 4.51 |
| How often is the pavement obstructed (n=1,367_FBA_) |  |  |  |  |
| None | 992 | 72.57 | 952 | 68.19 |
| Occasional | 283 | 20.70 | 312 | 22.35 |
| Often | 21 | 1.54 | 62 | 4.44 |
| N/A | 71 | 5.19 | 70 | 5.01 |
| Pavement width on the left hand side of road (n=1,358_FBA_) |  |  |  |  |
| None/narrow | 129 | 9.50 | 145 | 10.39 |
| 1 person/variable | 189 | 13.92 | 212 | 15.19 |
| 2 people | 910 | 67.01 | 869 | 62.25 |
| >2 people | 130 | 9.57 | 162 | 11.60 |
| N/A | 0 | 0.00 | 8 | 0.57 |
| Pavement width on the right hand side of road (n=1,353_FBA_) |  |  |  |  |
| None/narrow | 114 | 8.43 | 155 | 11.10 |
| 1 person/variable | 184 | 13.60 | 165 | 11.82 |
| 2 people | 918 | 67.85 | 897 | 64.26 |
| >2 people | 137 | 10.13 | 160 | 11.46 |
| N/A | 0 | 0.00 | 19 | 1.36 |
| How many pedestrians are on the segment (n=1,371_FBA_) |  |  |  |  |
| No people | 483 | 35.23 | 628 | 44.99 |
| Few | 837 | 61.05 | 651 | 46.63 |
| Many | 46 | 3.36 | 104 | 7.45 |
| Crowded | 5 | 0.36 | 2 | 0.14 |
| N/A | 0 | 0.00 | 11 | 0.79 |
| How many lanes are there (road types) (n=1,376_FBA_, n=1,393_GSV_) |  |  |  |  |
| One way | 26 | 1.89 | 32 | 2.30 |
| 2-way no lane marks | 1159 | 84.23 | 1096 | 78.68 |
| 2-way with lane marks | 172 | 12.50 | 240 | 17.23 |
| 3+ lanes | 11 | 0.80 | 4 | 0.29 |
| N/A | 8 | 0.58 | 21 | 1.51 |
| Road/Segment connectivity (n=1,388_FBA_) |  |  |  |  |
| Through road | 1033 | 74.42 | 1018 | 72.92 |
| Cul de Sac | 16 | 1.15 | 44 | 3.15 |
| Cul de Sac with pedestrian throughway | 335 | 24.14 | 312 | 22.35 |
| Pedestrianized | 4 | 0.29 | 5 | 0.36 |
| N/A | 0 | 0.00 | 17 | 1.22 |
| Does the segment have a traffic calming measure (n=1,371_FBA_) |  |  |  |  |
| Absent | 1170 | 85.34 | 1187 | 85.03 |
| At 1 or 2 points | 124 | 9.04 | 139 | 9.96 |
| Regularly | 72 | 5.25 | 58 | 4.15 |
| N/A | 5 | 0.36 | 12 | 0.86 |
| Type of parking available on the segment (n=1,378_FBA_) |  |  |  |  |
| None | 108 | 7.84 | 116 | 8.31 |
| Mostly restricted | 128 | 9.29 | 177 | 12.68 |
| Several sections of unrestricted | 179 | 12.99 | 286 | 20.49 |
| Unlimited | 955 | 69.30 | 804 | 57.59 |
| N/A | 8 | 0.58 | 13 | 0.93 |
| Amount of cars parked (n=1,385_FBA_) |  |  |  |  |
| None | 213 | 15.38 | 208 | 14.90 |
| Only a few | 883 | 63.75 | 946 | 67.77 |
| Bays half filled | 213 | 15.38 | 151 | 10.82 |
| Parking filled or obstruction of crossings | 72 | 5.20 | 66 | 4.73 |
| N/A | 4 | 0.29 | 25 | 1.79 |
| Amount of lamp posts (n=1,384_FBA_) |  |  |  |  |
| None | 52 | 3.76 | 58 | 4.15 |
| Sporadically / Damaged | 114 | 8.24 | 39 | 2.79 |
| Regular on one side | 348 | 25.14 | 559 | 40.04 |
| Regular on both sides | 870 | 62.86 | 708 | 50.72 |
| N/A | 0 | 0.00 | 32 | 2.29 |
| Is the segment on a slope (n=1,382_FBA_) |  |  |  |  |
| Flat | 877 | 63.46 | 733 | 52.51 |
| Slight hill | 420 | 30.39 | 488 | 34.96 |
| Steep hill | 85 | 6.15 | 166 | 11.89 |
| N/A | 0 | 0.00 | 9 | 0.64 |
| ***Aesthetics*** |  |  |  |  |
| Neighbourhood watch (n=1,375_FBA_, n=1,395_GSV_) |  |  |  |  |
| None | 611 | 44.44 | 948 | 67.96 |
| Few | 629 | 45.75 | 347 | 24.87 |
| Many | 135 | 9.82 | 93 | 6.67 |
| N/A | 0 | 0.00 | 7 | 0.50 |
| Security measures (n=1,375_FBA_, n=1,395_GSV_) |  |  |  |  |
| None | 357 | 25.96 | 213 | 15.27 |
| Few | 875 | 63.64 | 961 | 68.89 |
| Many | 143 | 10.40 | 212 | 15.20 |
| N/A | 0 | 0.00 | 9 | 0.65 |
| Greenery (n=1,377_FBA_, n=1,395_GSV_) |  |  |  |  |
| None | 46 | 3.34 | 40 | 2.87 |
| Little | 453 | 32.90 | 410 | 29.39 |
| Moderate | 611 | 44.37 | 711 | 50.97 |
| Extensive | 267 | 19.39 | 221 | 15.84 |
| N/A | 0 | 0.00 | 13 | 0.93 |
| Graffiti (n=1,373_FBA_, n=1,395_GSV_) |  |  |  |  |
| None | 1212 | 88.27 | 1365 | 97.85 |
| Little | 138 | 10.05 | 19 | 1.36 |
| Moderate | 21 | 1.53 | 2 | 0.14 |
| Extensive | 2 | 0.15 | 0 | 0.00 |
| N/A | 0 | 0.00 | 9 | 0.65 |
| ***Land use*** |  |  |  |  |
| Predominant land use (n=1,351_FBA_, n=1,395_GSV_) |  |  |  |  |
| Purpose built block of flats | 51 | 3.77 | 40 | 2.87 |
| Offices | 5 | 0.37 | 4 | 0.29 |
| Shops and services, schools | 45 | 3.33 | 41 | 2.94 |
| Offices / shops with flats above | 6 | 0.44 | 17 | 1.22 |
| Industrial / Other commercial buildings / car parks | 49 | 3.63 | 46 | 3.30 |
| Terraced houses | 216 | 15.99 | 250 | 17.92 |
| Detached or semi-detached houses | 895 | 66.25 | 894 | 64.09 |
| Open green areas | 79 | 5.85 | 91 | 6.52 |
| Derelict or vacant building/plot | 5 | 0.37 | 2 | 0.14 |
| N/A | 0 | 0.00 | 10 | 0.72 |
| Secondary land use (n=823_FBA_, n=1,389_GSV_) |  |  |  |  |
| Purpose built block of flats | 55 | 6.68 | 65 | 4.68 |
| Offices | 11 | 1.34 | 2 | 0.14 |
| Shops and services, schools | 117 | 14.22 | 114 | 8.21 |
| Offices / shops with flats above | 19 | 2.31 | 41 | 2.95 |
| Industrial / Other commercial buildings / car parks | 58 | 7.05 | 72 | 5.18 |
| Terraced houses | 110 | 13.37 | 161 | 11.59 |
| Detached or semi-detached houses | 142 | 17.25 | 167 | 12.02 |
| Open green areas | 292 | 35.48 | 212 | 15.26 |
| Derelict or vacant building/plot | 19 | 2.31 | 11 | 0.79 |
| N/A | 0 | 0.00 | 544 | 39.16 |
| ***Quality issues with GSV*** |  |  |  |  |
| Lighting |  |  | 2 | 0.14 |
| Shadows |  |  | 3 | 0.21 |
| Weather |  |  | 49 | 3.51 |
| Obstructions |  |  | 41 | 2.94 |
| Skipping |  |  | 96 | 6.88 |

^a^ FBA represents the number of segments in foot based audits, GSV represents the number of segments in google street view audits.

FBA: Foot-based audits; GSV: Google Street View; N/A: Not available; SD: Standard deviation.
